# Identifying systemic risks and mitigation strategies of artificial intelligence in agriculture: from social-technical-ecological systems framework

**DOI:** 10.3389/fpls.2026.1811551

**Published:** 2026-06-05

**Authors:** Yuyang Yuan, Yong Sun

**Affiliations:** School of Public Administration, Guangzhou University, Guangzhou, China

**Keywords:** AI in agriculture, artificial intelligence, intelligent phytoprotection, risk analysis, smart agriculture, sustainable agriculture

## Abstract

While the transformative potential of Artificial Intelligence (AI) in global agriculture is widely acknowledged, especially its contributions to plant protection and agricultural production, much of the research mainly highlights its benefits, overlooking the potential impacts of AI on agricultural systems, including planting, cropping, irrigation, and fertilization. While certain studies have started to explore specific challenges, a comprehensive and integrated analysis of these risks across agricultural systems remains largely unaddressed. This study employs a narrative review and in-depth reflection, adopts the Social-Technical-Ecological Systems (STES) framework to analyze these risks, with plant protection and development as the illustrative examples. The social subsystem faces potential risks, including unemployment, social inequality, and systemic exclusion. Within the technical subsystem, we identify risks such as uncertainties in technical devices, inaccuracies in AI model decisions, untraceable AI black-box decision-making, and network security vulnerabilities. Within the ecological subsystem, AI may lead to biodiversity loss, climate uncertainties, and potential environmental pollution. To mitigate these risks, we propose targeted strategies. In the social subsystem, recommendations include enhancing farmers’ livelihood resilience, improving the inclusivity and accessibility of AI, and integrating principles of social equity. In the technical subsystem, this involves optimizing AI agricultural devices, enhancing the accuracy of AI decision-making, improving the transparency of AI models, and ensuring network security. For the ecological subsystem, strategies focus on embedding biodiversity goals, developing climate-friendly AI agriculture, and integrating ecological monitoring and evaluation. At the overall system level, if the balance among subsystems is not sufficiently considered, it may lead to cross-system risks. Collaborative risk governance is crucial for balancing social equity, technical efficiency, and ecological sustainability. This study provides actionable guidance for policymakers, AI developers, and farmers to achieve efficient, equitable, and sustainable AI-driven agriculture, offering important reference value for advancing intelligent phytoprotection and smart agricultural development.

## 1 Introduction

With the rapid development of Artificial Intelligence (AI) technologies, many countries and regions worldwide are beginning to explore the application of AI technologies in agricultural systems, such as plant protection, crop development, food production, and related fields. The definition of AI is often associated with the computer science methods it employs, such as machine learning (ML), reinforcement learning (RL), and deep learning (DL). Additionally, AI software can also take the form of robotic arms, robots, and other similar devices (Gardezi et al., 2024). These AI technologies are gradually transforming agricultural production processes such as planting, cropping, fertilizing, and irrigating.

The significant benefits of AI in agriculture are unquestionable. AI can significantly empower agricultural production and plant recognition (Han et al., 2025b), help farmers make agricultural decisions, promote sustainable protection of plants, and share the benefits from AI-driven agriculture (Klerkx et al., 2019; Omotayo et al., 2025). Numerous real-world cases have shown the positive value of AI in agriculture. For instance, AI in agriculture can effectively enhance farmers’ decision-making capabilities and agricultural production efficiency, thereby strengthening the resilience of sustainable livelihoods for farmers (Yuan and Sun, 2025). Through AI technologies such as machine learning and computer vision, the large-scale production efficiency of biological control agents can be significantly enhanced, thereby solving pest infestation issues in modern agriculture (Javed et al., 2025). Applying ML and predictive analytics algorithms in agriculture can significantly increase crop yields, reduce losses, and promote food security (Rugji et al., 2025). Currently, more and more countries are adopting AI in agriculture, so as to enhance agricultural efficiency and promote intelligent phytoprotection. For example, China is speeding up the digital and intelligent transformation of agriculture.

Currently, AI in agriculture is gaining widespread attention globally, reshaping the development model of agriculture worldwide (Madhuri et al., 2025). The researchers have increasingly focused on the academic topics related to AI in agriculture in recent years. In this context, scholars have particularly focused on the positive functions and values of AI in agriculture, including intelligent phytoprotection, increased production efficiency, biodiversity, and other areas. Unlocking the value of AI in enhancing agricultural productivity and providing precise agricultural decisions. Existing research generally indicates that the practices of AI in agriculture are helpful to make precision agricultural decisions, optimize the management of agricultural resources, and elevate overall agricultural productivity (Arevalo-Royo et al., 2025; Nayal et al., 2022; Rugji et al., 2025; Singh and Goyal, 2023). AI is widely regarded as the most promising technology for avoiding the global agricultural production crisis (Kazancoglu et al., 2024). Nevertheless, as research progresses and deepens, AI in agriculture has begun to reveal and highlight some unintended negative issues in real-world applications.

Recently, some research has begun to pay attention to the risks from AI in agriculture. For instance, some studies indicate that although the use of AI in agriculture has gained significant benefits, it also brings significant risks to human safety, the environment, and society, including device uncertainty, algorithmic bias, security threats, and so on (Galaz et al., 2021; Gao et al., 2024; Lacey, 2020; Sparrow et al., 2021; Stodle et al., 2025). These risks not only directly harm the agricultural system but also reduce users’ trust, ultimately limiting the further development of AI in agriculture (Barbosa et al., 2020; Dixit et al., 2023). There is an important need to increase attention and research on the risks associated with AI in agriculture. While existing studies provide us with some inspiration, however, there remain some research gaps: 1) Overall, existing research has tended to focus more on the positive values of AI, showing a certain technological determinism. The quantity and quality of research around mitigating risks associated with AI in agriculture require further enhancement. 2) Although existing research recognizes the risks associated with AI in agriculture, it often focuses on a single technical dimension (Lacueva-Perez et al., 2025), social dimension (Gardezi et al., 2024), or ecological dimension (Sajith et al., 2022), or simply lists the complex risks of AI in agriculture (Sparrow et al., 2021), lacks systematic summarization, with no research consensus established (Regan, 2019). 3) Finally, the absence of a forward-looking system of strategies for the risks from AI in agriculture, and lack of a comprehensive governance framework, it cannot provide scientific guidance for practice.

**Figure 1:**
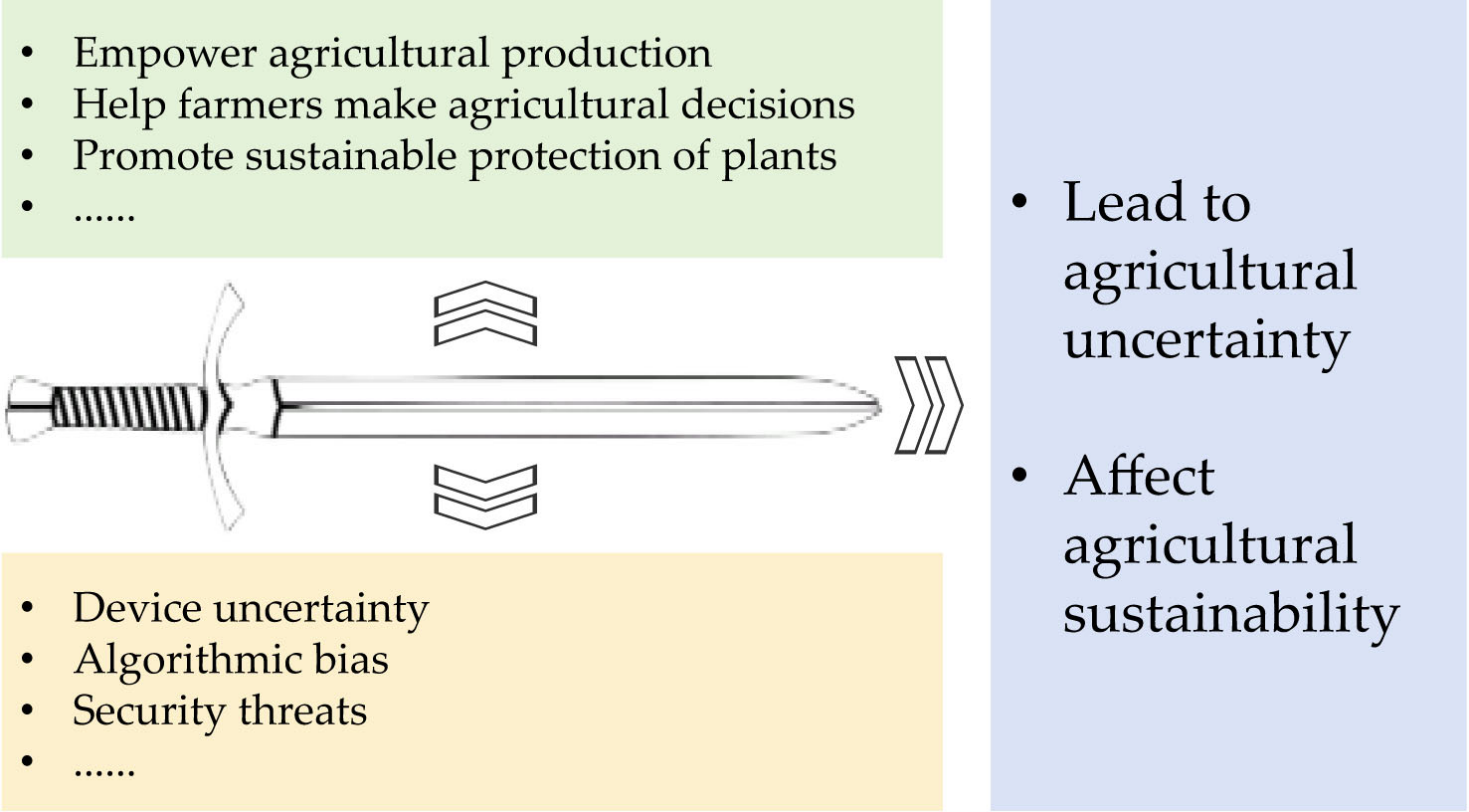
The double-edged sword effect of AI in agriculture.

Based on a review of relevant research and a reflection on risk society theory (Beck, 2011), one of the basic characteristics of modern society is risk. While modern developments bring positive benefits, they also bring certain risks (Mythen, 2021), AI in agriculture is no exception. It is essential to clearly recognize that AI in agriculture shows a significant double-edged sword effect, including in the context of plant protection and development. If this effect is not taken seriously, it may lead to agricultural uncertainty and affect agricultural sustainability (**Figure 1**). In the development of AI in agriculture, it is essential to fully consider the systemic risks it may face. To this end, we must actively respond to challenges in data collection, model training, and other critical stages, try to prevent and avoid potential negative impacts from AI, and promote the sustainable development of AI in agriculture (Leal Filho and Gbaguidi, 2024; Li et al., 2024; Tzachor et al., 2022).

To address these gaps, our study focuses on the risks of AI in agriculture. We adopt a narrative review methodology, and conduct a deep analysis of 125 relevant literatures from the Web of Science and Scopus. Our primary goals are to systematically categorize and summarize the diverse potential risks faced by AI in agriculture, and provide reasonable risk management strategies to promote plant protection, food production, and related objectives.

Our paper’s contributions: 1) enhancing awareness of risks associated with AI in agriculture. While existing research generally focuses on the positive values of AI in agriculture, it is essential to fully recognize the negative effects of AI in agriculture, avoid potential risks, and promote the sustainable development of AI in agriculture (Gao et al., 2024). It should be noted that plants, as the core of agricultural systems, this paper focuses specifically on the risks associated with the use of AI in plant protection scenarios, such as pest and disease identification, fertilizer management, and the preservation of plant diversity. 2) Understanding the systemic risks of AI in agriculture comprehensively. To this end, this study attempts to adopt the Social-Technical-Ecological Systems (STES) framework, and identify and categorize the systemic risks of AI in agriculture from a wholeness perspective. 3) Establishing a comprehensive risk mitigation framework for AI in agriculture. By systematically summarizing and categorizing risks from AI in agriculture, this study proposes effective strategies to mitigate various risks. It provides a forward-looking governance framework and offers insights for risk mitigation policies to inform the future development of AI in agriculture, particularly in plant protection and crop development, thereby promoting global food production and sustainable agricultural practices.

## 2 Materials, methods, and framework

### 2.1 Materials

The analytical materials for this review are sourced from the core collections of two premier academic databases: Web of Science (WoS) and Scopus. The new field of AI in agriculture has produced a large amount of literature of varying quality, posing a significant challenge for identifying high-impact, rigorous research. To ensure the authority and comprehensiveness of our review, it is necessary to rely on globally recognized and meticulously curated sources.

The Web of Science Core Collection, especially the SCI, SSCI, and A&HCI in it, is renowned for its strict journal selection standards and concentration of high-impact publications. In addition, Scopus offers broader coverage, particularly of emerging research areas, and international publications, serving as an important complement to Web of Science. By using these two databases, we conducted a more comprehensive literature search, thereby reducing potential selection bias and minimizing the risk of missing key studies. Literature indexed in these core collections is generally characterized by frontier topics, scientific methodologies, and robust conclusions. Therefore, this dual-database strategy supports the breadth, quality, and reliability of our research content and findings.

We employed a scientific approach to screen the literature set included in the analysis. (1) We prioritize the SCI, SSCI, AHCI core collection in WoS, then explore other literature with high download or citation counts in WoS and Scopus. (2) We select research articles and review articles in English. (3) Select literature that is directly or indirectly related to the risks of AI in agriculture. (4) Inclusion of articles employing a scientific research methodology and yielding reliable results. Through the above methods, the dataset for this study was formed (**Table 1**).

**Table 1:** Inclusion and exclusion criteria.

Criterion	Inclusion	Exclusion
Database type	Primarily SCI, SSCI, AHCI, as well as other literature with high download or citation counts	Other types
Language	English	Non-English
Publication type	Research articles and review articles	Other types
Relevance to research topic	Articles related to the risks of AI in agriculture	Articles not related to the risks of AI in agriculture
Methodological quality	Articles with scientific and clear methodologies	Articles with methodological flaws, such as inadequate sample size and bias in data selection
Research findings	Clear and comprehensive findings	Inadequate or unclear findings

### 2.2 Methods

Our study applies a narrative review approach, combined with in-depth reflection, to analyze the systemic risks of AI in agriculture and provide reasonable risk mitigation strategies. To ensure transparency, reproducibility, and methodological rigor in the research, the review process referred to the principles of the Preferred Reporting Items for Systematic Reviews and Meta-Analyses (PRISMA 2020) guidelines. This process, including a clear search strategy and explicit screening criteria, was applied to guarantee the comprehensive and scientific nature of the literature dataset supporting the narrative analysis. The narrative review approach was chosen for its flexibility in synthesizing diverse cross-disciplinary literature. It is particularly well-suited for comprehensively summarizing and constructing theories around interdisciplinary, complex, and intersecting topics, while also enabling in-depth analysis of risk forms and governance strategies. The study primarily consists of two stages: 1) literature search and screening; 2) content analysis and synthesis.

The first stage involved a literature search and screening process. We constructed a thematic search strategy around three conceptual dimensions. The first dimension, AI technologies, which are related to computer science methods (Gardezi et al., 2024), mainly included the terms: “AI”, “Artificial Intelligence”, “machine learning”, “deep learning”, “neural networks”, “computer vision”, “robotics”, “automation”, and “smart systems”. The second dimension, agricultural contexts, mainly including: “agriculture”, “farming”, “crop production”, “precision agriculture”, “smart farming”, “livestock management”, and “irrigation”. The third dimension, risks, mainly including: “risk”, “challenge”, “threat”, “vulnerability”, “security”, “ethical issues”, “bias”, “privacy”, and “environmental impact”. To ensure the precision and quality of the search, strict inclusion criteria were applied: the search was confined to the Web of Science and Scopus databases; search was conducted at the topic level; only research articles and reviews were considered; and the language was restricted to English. The initial search, performed on January 10, 2026, retrieved 11,107 records from Scopus and 10,401 from Web of Science. After removing duplicates, 16,710 unique publications remained for further screening.

**Figure 2:**
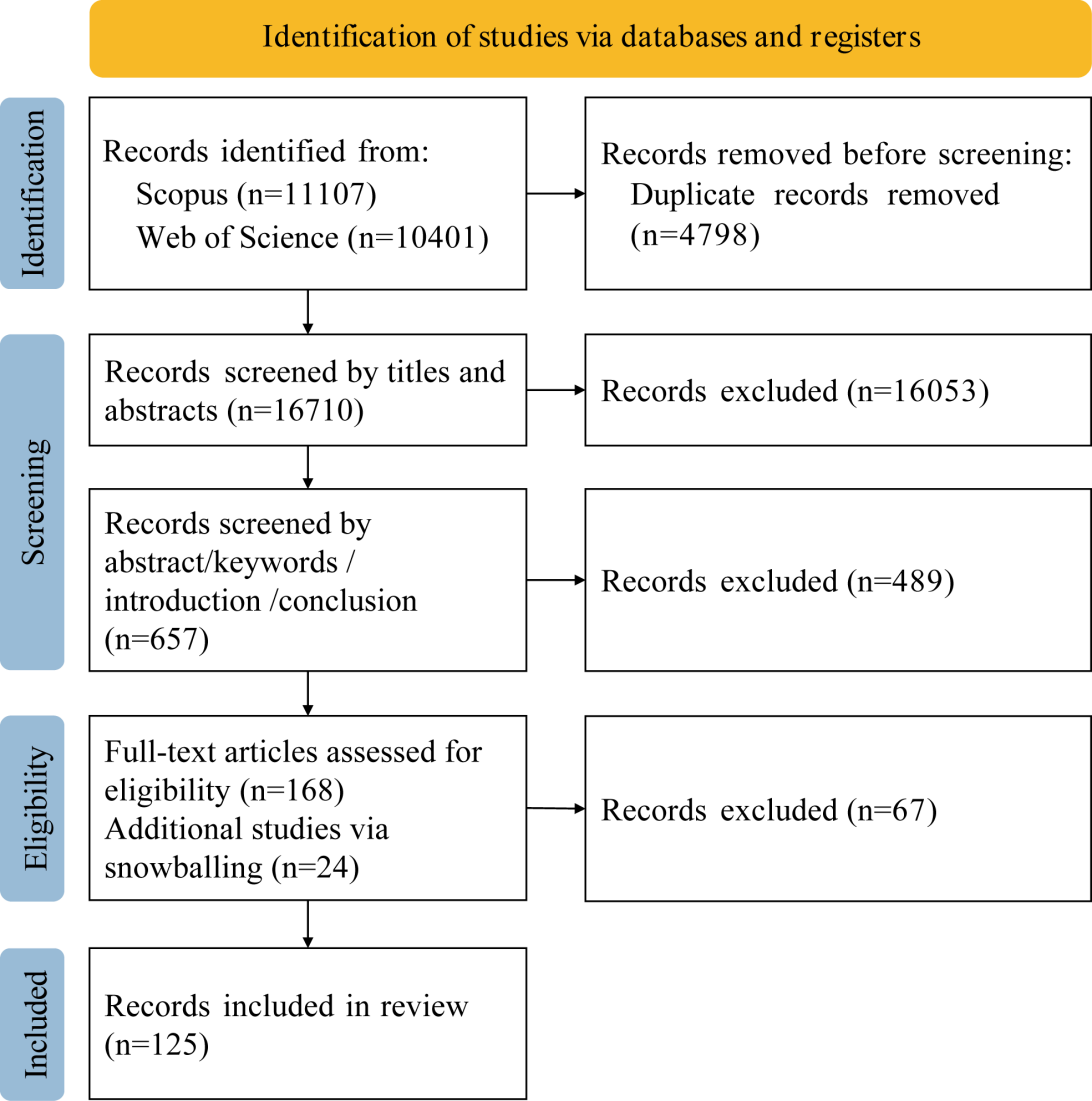
PRISMA approach for identification and selection of studies.

The second stage involved a two-step systematic screening process to ensure both methodological quality and thematic relevance. During the initial screening, we conducted relevance sorting of article titles and abstracts to exclude studies that either failed to address the risks from AI-driven agriculture or focused only on technical applications without examining broader social and economic impacts. This step eliminated 16,053 irrelevant records, leaving 657 articles that proceeded to the secondary screening. Then, the secondary screening entailed a rapid assessment of abstracts, keywords, introductions, and conclusions to verify thematic relevance with the review’s scope. This process further narrowed the selection to 168 highly relevant articles, which were used for full-text evaluation. In this full-text assessment, we conducted a comprehensive evaluation of methodological rigor, logical coherence, and argument relevance to confirm their suitability for inclusion. During this process, 67 articles were excluded for failing to meet the inclusion criteria, while 101 articles qualified for the core analysis set. Furthermore, we supplemented the core set of studies with some additional articles identified through forward and backward snowballing, and incorporated further relevant studies during manuscript revision to capture the latest developments and address evidence gaps, a total of 24 articles. Ultimately, this systematic selection process yielded 125 high-quality articles, which form the analytical foundation of this review (see **Figure 2**).

The third stage of our research focused on content analysis and synthesis. To use an analytical framework and ensure research depth, we adopted the STES framework. Based on this framework, we conducted a structured inductive thematic analysis to derive the identified AI risks and mitigation strategies from the 125 included articles. Adopting a two-cycle coding framework, we first performed open coding to extract all relevant statements on risks and mitigation strategies from the full text, generating initial descriptive codes. We then combined these codes into coherent, higher-order themes through axial coding. This analytical approach ensures that the final themes are rigorously grounded in the literature, enhancing the credibility of our review’s findings.

### 2.3 Analytical framework

The core of AI in agriculture is to systematically integrate a series of modern AI technologies into the agricultural system, driving the transformation of agricultural decision-making from people to algorithmic approaches. Enable AI to directly determine or assist precision decision-making across all stages of agricultural production, including irrigation, planting, fertilization, and sales, thereby reshaping the agricultural system (Essenfelder et al., 2025). However, while AI is driving agricultural transformation, it also raises some issues and uncertainty (Omotayo et al., 2025).

Some studies have indicated that AI systems are characterized by high complexity. Applying AI technologies to agriculture can be viewed as adding more connections to complex Socio-Ecological and Socio-Technical Systems, thereby further increasing the complexity of agricultural systems (Galaz et al., 2021; Mmbando, 2025). The application of AI technology will have an impact on society and ecology. Through the synthesis of similar studies and analysis of relevant theories, it has been found that AI in agriculture primarily has direct impacts on social systems, technical systems, and ecological systems.

Previous studies have already used the Social-Technical-Ecological Systems theory or Social-Technical-Environmental Systems theory to conduct comprehensive analyses of specific issues, providing inspiration for the analytical framework of this paper. For instance, research has shown that integrating technology into social-ecological systems can construct the STES framework (Ahlborg et al., 2019). The framework has been previously applied to analyze the development of urban circular economies (van der Leer et al., 2018). Similarly, the Social-Technical-Environmental Systems analytical approach has been applied in studies concerning the enhancement of wind energy systems through the utilization of biomass energy storage technology (Denholm, 2006). Furthermore, the Social-Technical-Environmental Systems perspective has been applied in research surrounding resilience theory. In this context, the social systems include factors such as social structures, economics, and politics; the technical systems include elements like infrastructure; and the ecological systems include climate and the biosphere (Tamberg et al., 2022). The Social-Technical-Environmental Systems framework has also been applied to discussions regarding energy efficiency improvements in the commercial and residential sectors (Kumar et al., 2020). The Social-Technical-Ecological Systems theory is similar to the Social-Technical-Environmental Systems theory. Based on existing research and the summary of the relevant literature, we adopted the Social-Technical-Ecological Systems as the analytical framework.

Based on the STES, AI technology not only influences the operation of the agricultural technical system, but also deeply impacts human society, including social equity and social culture. Furthermore, the technical systems also have internal links to the ecological systems. The development of AI in agriculture is a typical human technological application activity that involves energy consumption and ecological planning adjustments. It will impact the ecological system. Evidently, the STES can fundamentally address issues related to technical embedding, and provide a comprehensive analytical framework specifically designed to assess the risks related to AI in agriculture. Nevertheless, the STES framework is an effective framework for integrating multidisciplinary perspectives (Lin et al., 2025).

Therefore, from the STES perspective, AI in agriculture is a complex systemic thing, and may face various risks from technical systems, social systems, and ecological systems. Each subsystem may face certain risks. For the technical subsystem, risks are primarily manifested at the level of technical uncertainty, which arise from issues within the technology itself, including device aging, software bugs, and network threats. For the social subsystem, risks primarily manifest in the negative impact of AI on rural society, such as AI-driven labor displacement, socio-economic inequality, and systemic exclusion. Finally, the ecological subsystem risks are unanticipated environmental problems arising from the application of AI in agriculture, such as erroneous decisions leading to excessive fertilization or over-pesticide application, which can disrupt the ecological balance. Furthermore, the balance among the three subsystems cannot be overlooked, as it may lead to unintended cross-system risks. We analyze the risks from AI in agriculture within the social subsystem, the technical subsystem, and the ecological subsystem one by one. Then, we comprehensively integrate the risks facing the overall STES. Thus, we can fully understand all the risks that come with AI in agriculture.

## 3 Systemic risks of AI in agriculture

**Figure 3:**
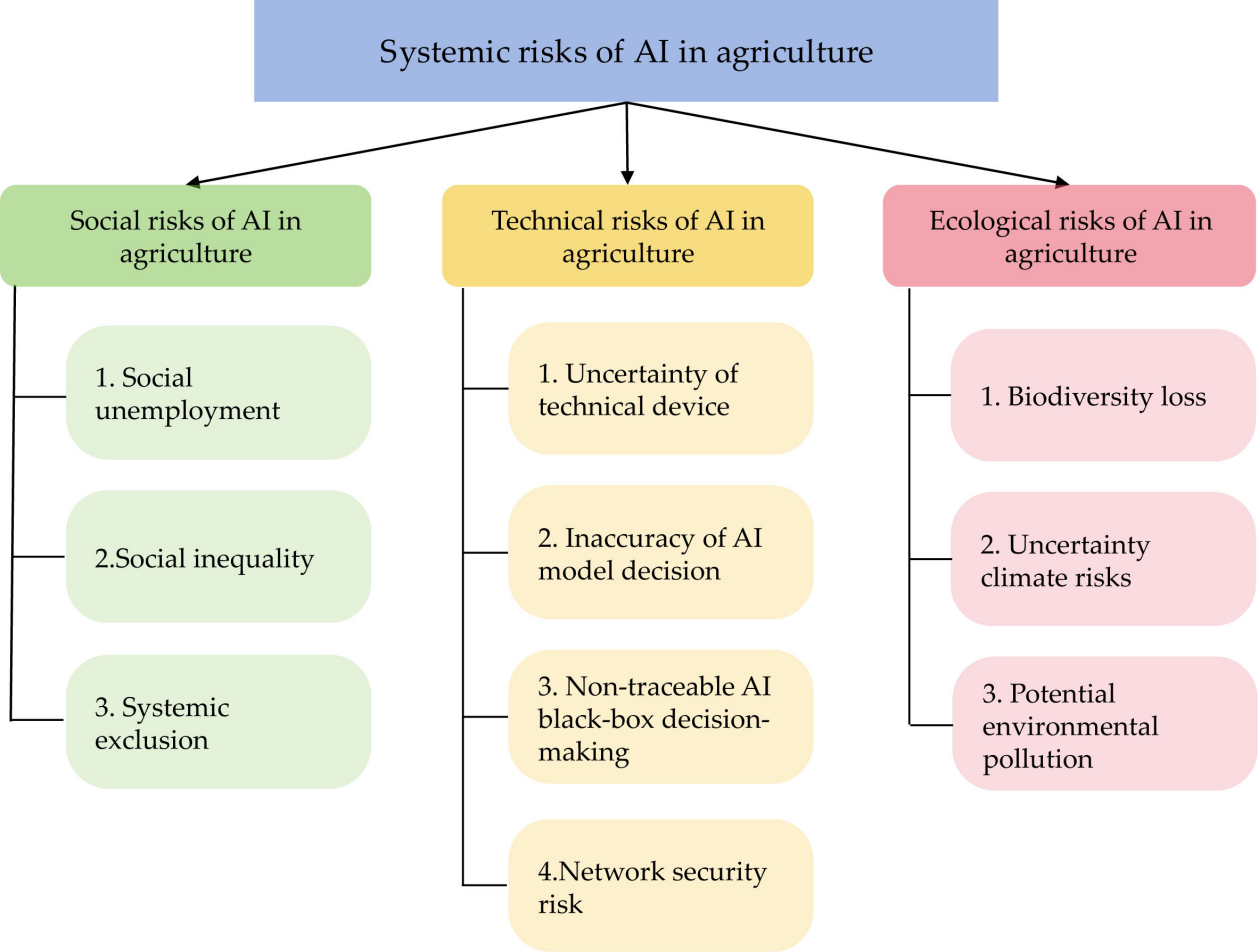
Systemic risks of AI in agriculture.

AI in agriculture, including using AI in planting, cropping, and other agricultural production, faces various forms of risk across its technical, social, and ecological subsystems. The key risks associated with these three subsystems are shown in **Figure 3**.

### 3.1 Social risks of AI in agriculture

AI in agriculture faces risks at the social subsystem level, including unemployment risks of AI replacing human labor, increased inequality due to socioeconomic barriers to AI adoption, and systemic exclusion caused by algorithmic bias (**Table 2**).

**Table 2:** Overview of social risks of AI in agriculture.

Subsystem	Risk category	Key manifestation	Representative references
Social	Social unemployment	AI-driven automation replaces agricultural labor, threatening the livelihoods of farmers and workers in agriculture-related sectors	Leal Filho and Gbaguidi, 2024; Sandbrook, 2025; Gonzalez-Rodriguez et al., 2024
	Social inequality	Unequal access to AI technologies widens the gap between large-scale and smallholder farmers	Bozeman et al., 2024; Khanna et al., 2024; Kalimuthu et al., 2024
	Systemic exclusion	Algorithmic bias and data bias cause AI models to overlook smallholders and marginalized groups	Gardezi et al., 2024; Li et al., 2024

#### 3.1.1 Social unemployment

Agriculture is a major field of employment in most countries (Raghuvanshi et al., 2022). With the widespread adoption of AI in agriculture, unmanned technologies are gradually replacing farmers, leading to significant social issues such as agricultural labor surplus and farmer unemployment (Leal Filho and Gbaguidi, 2024; Sandbrook, 2025). Currently, highly efficient unmanned technologies, including smart autonomous tractors, drones, and robots, are more and more important in agricultural production. These technologies are gradually replacing traditional agricultural labor, creating a threat to farmers’ livelihoods (Gonzalez-Rodriguez et al., 2024; Sparrow et al., 2021). Taking the field of plant protection and development as examples. An autonomous weeding system used in orchards can reduce reliance on manual labor (Han et al., 2025a), which may lead to a reduced demand for workers in orchards. Jobs such as plant protection technicians and plant quarantine inspectors are also facing the possibility of replacement by AI technologies like neural networks. More seriously, the unemployment issues caused by AI replacing human labor have already arisen in populist movements across Europe (Dorigo et al., 2025).

#### 3.1.2 Social inequality

From the perspective of socio-economic stratification, AI in agriculture requires significant economic investment and professional knowledge related to AI. Not all agricultural producers can equally have access to and share in the benefits of AI development, which may increase social inequality (Bozeman et al., 2024; Lakhiar et al., 2025; Pimenow et al., 2025). At present, due to the relatively low digital literacy and income levels in some rural areas and among farming communities, especially smallholder farmers (Yuan and Sun, 2024), they face difficulties in accessing and applying modern AI technologies. This may lead to a widening wealth gap between large-scale and small-scale farmers (Khanna et al., 2024; Li et al., 2024; Maraveas et al., 2022). At the same time, this will further widen the gap in agricultural development between high-income and low-income countries (Kalimuthu et al., 2024), increasing global agricultural inequality.

In addition, farmers often lack sufficient understanding of AI clauses, placing them in a disadvantaged position of information asymmetry. This may lead to potential harm to their interests and widen the gap between different users (Fuentes-Penailillo et al., 2024; Ibrahim and Truby, 2023). AI in agriculture may also bring about issues such as widening wealth gaps and wealth monopolization between groups of different socioeconomic status, increasing socioeconomic inequality, and threatening social stability (Sandbrook, 2025; Sparrow et al., 2021). For example, farmers with better financial resources can fully deploy AI-powered plant protection equipment, thereby significantly improving pest and disease control, increasing irrigation efficiency, and reducing crop losses, whereas smallholders often cannot afford the high-cost AI equipment. This inequality in access to technology tends to widen the gap in crop yields between farmers of different scales.

#### 3.1.3 Systemic exclusion

It is worth noting that human society itself has certain biases, and these biases are easily copied into AI models. This leads to issues such as data bias and algorithmic bias, causing AI to potentially generate biased decisions that increase exclusion and undermine social fairness (Bozeman et al., 2024; Gardezi et al., 2024; Leal Filho and Gbaguidi, 2024; Li et al., 2024). For example, during the initial training phase, AI pest and disease detection models tend to rely primarily on data from mainstream crops, such as wheat and rice. As a result, data on diseases affecting non-mainstream crops like buckwheat and quinoa, which are grown by some farmers, may be overlooked. This means that farmers growing mainstream crops benefit more from AI pest detection models, while those growing non-mainstream crops may face systemic exclusion.

### 3.2 Technical risks of AI in agriculture

AI technology brings a range of positive values, such as precision, scientific, and high efficiency. Theoretically, these features are conducive to enhancing plant protection, optimizing agricultural decision-making, improving production output, and agricultural sustainability. However, AI is not omnipotent. Its application in real-world agricultural scenarios may face risks, such as uncertainty of technical devices, inaccurate decision-making, lack of decision traceability, and network security threats (**Table 3**).

**Table 3:** Overview of technical risks of AI in agriculture.

Subsystem	Risk category	Key manifestation	Representative references
Technical	Uncertainty of technical devices	Sensors, drones, and other AI-related devices face wear, malfunction, and signal instability in farmland environment	Visser et al., 2021; Galaz et al., 2021; Kumar and Patel, 2023
	Inaccuracy of AI model decisions	Insufficient or biased training data leads to inaccurate decision-making and poor adaptability	Mittal et al., 2024; Gupta and Tripathi, 2024; Anandhakrishnan and Jaisakthi, 2022
	Non-traceable black-box decision-making	Lack of transparency in AI algorithms makes it difficult for users to verify decisions	Izquierdo-Bueno et al., 2024; Stodle et al., 2025; Sandbrook, 2025
	Network security risk	AI systems in open networks are vulnerable to cyber-attacks, and user privacy data breaches	Gao et al., 2024; Hazrati et al., 2022; Bui et al., 2024

#### 3.2.1 Uncertainty of technical device

AI in agriculture involves the integration of large AI models with a range of hardware devices, such as sensors, remote sensing, robotic arms, robots, and drones (Gardezi et al., 2024). Specifically, it relies heavily on hardware devices such as sensors and remote sensing to monitor and record various agricultural environmental data, including temperature, humidity, and soil fertility, then generates precise agricultural decisions through AI large models. However, devices such as sensors are exposed to agricultural environments with long-term humidity, intense sunlight, and rainfall. This causes significant wear and damage to electronic components and machinery. Research has summarized a series of data and case studies on device failures. For instance, sensors operating in muddy and humid farmland conditions may lead to inaccurate recordings of plant growth and crop health, while agricultural AI algorithms can produce errors during extreme weather fluctuations. Research shows that the error rate of GPS-based yield monitors is over 10% (Visser et al., 2021).

Consequently, AI technical devices in the agricultural environment inevitably face uncertainty risks, such as short circuits, hardware aging, internal malfunctions, and sudden failure, any of which can lead to the disruption of agricultural production (Galaz et al., 2021; Visser et al., 2021). For instance, sensors deployed to monitor plant growth and detect diseases may produce erroneous signals due to environmental interference such as waterlogging or insect attachment on the foliage, which can lead AI systems to misjudge plant health and subsequently generate inappropriate management recommendations. Currently, early warning alerts regarding plant pests and diseases generated by AI systems are primarily disseminated to farmers via SMS. However, signal instability and uncertainty in rural and agricultural environments often lead to message loss, preventing farmers from receiving early warning messages in a timely manner (A. Kumar and Patel, 2023).

#### 3.2.2 Inaccuracy of AI model decision

1. Data training limitations bring risks of inaccurate decision-making. AI empowers agricultural production mainly by providing users with accurate agricultural decision-making. However, it is difficult for AI’s decision-making to maintain 100% accuracy, and its effectiveness is highly dependent on the completeness of the training data. The more comprehensive and extensive the training data is for the AI model, the more accurate the decisions it generates, and vice versa (Essenfelder et al., 2025; Mittal et al., 2024; Sparrow et al., 2021). Due to factors such as geographical complexity and changes in external agricultural environments, much agricultural data remains inaccessible. This frequently leads to challenges for AI models, including insufficient training data and poor data quality, reducing the accuracy of AI decision-making (Ali et al., 2024; Chen et al., 2021; Kpodo and Nejadhashemi, 2025).

When applying AI to agricultural production, the inaccuracy of decision-making always occurs. For example, the low resolution of Sentinel-2 satellite imagery makes it difficult to capture small-scale vineyard land data (Lacueva-Perez et al., 2025); training datasets for AI-based crop disease identification are often of poor quality and incompatible across platforms; AI systems struggle to learn from sufficient data, thereby compromising decision accuracy (Gupta and Tripathi, 2024); and an Indian machine learning model for rice production failed to incorporate the duration data of extreme weather events, leading to insufficient consideration during decision-making (Bowden et al., 2025). All of these factors bring risks of inaccurate decision-making. Additionally, AI models require continuous training on new data, so that models can optimize and upgrade their capabilities. Otherwise, they will generate decisions based on old data, failing to adapt to new planting scenarios and agricultural environments, leading to inaccurate decision-making (Kikon and Deka, 2022; Peters et al., 2020).

2 Heterogeneous agricultural contexts reduce the effectiveness of general-purpose AI models. Popular AI models on the market are often referred to as general-purpose AI, indicating that the same AI can frequently be applied across a wide range of different scenarios. However, specific AI models are often trained using agricultural data from particular regions, which may differ significantly from the real agricultural environments in other areas. It leads to general-purpose AI models failing to achieve 100% accuracy when applied elsewhere, often resulting in underfitting or overfitting issues that compromise the precision of agricultural decision-making (Anandhakrishnan and Jaisakthi, 2022; Gautron et al., 2022; Goyal et al., 2025).

It is essential that AI in agriculture gives careful consideration to regional differences, recognizing the limitations of AI models in diverse local agricultural contexts (Gautron et al., 2022; Hughes et al., 2022; Zewdu et al., 2025). For example, research has found that when neural network models with over 98% prediction accuracy are applied to new agricultural environments, they may generate erroneous decisions regarding planting, fertilization, irrigation, and other crop production practices (Kumar et al., 2022). General-purpose AI models trained in the agricultural context characterized by “few people and much land” will face limitations when applied to other regions characterized by “much people and few land,” as well as areas with different climate conditions (Quddus et al., 2025).

#### 3.2.3 Non-traceable AI black-box decision-making

Many AI technologies, such as algorithms, machine learning, and deep learning, lack transparency during running, which is described as the black-box (Buyuktepe et al., 2025; Izquierdo-Bueno et al., 2024; Oikonomidis et al., 2023). AI users always find it difficult to understand and verify the validity of the outputs. More seriously, AI illusions and faked data have become increasingly significant issues, as some AI models potentially generate agricultural advice containing false information (Dorigo et al., 2025). Once AI models generate misleading decisions, users find it difficult to understand and identify potential errors. If there is a lack of professional review or the inability to identify issues based on one’s own expertise, it may lead users to make error decisions (Sandbrook, 2025; Stodle et al., 2025). In this context, it is easy to lead to technological runaway and risks of untraceable liability, thereby causing damage to user benefits. For instance, an AI-driven crop irrigation system suddenly delivered excessive water, causing root rot and reduced yields. However, due to the system’s lack of transparency, farmers were unable to determine whether the issue stemmed from sensor malfunction or algorithmic error, leaving them with no recourse for accountability.

#### 3.2.4 Network security risk

AI in agriculture has reshaped farming models and planting methods, making agricultural producers highly reliant on technologies such as large-scale models, big data, and machine learning to conduct agricultural production. Operating in an open network environment, users may face risks of network attacks and privacy breaches (Bui et al., 2024; Kumar et al., 2021).

1. Network Attack Risks. The use of AI puts users in the digital world, where they manage agricultural production and plant management by operating various devices and monitoring data. This makes them easily face hacker attacks, highlighting the need to pay more attention to network security issues of AI in agriculture (Sparrow et al., 2021). Various agricultural technologies such as large-scale models, big data, agricultural robots, and autonomous tractors all require network support, making them likely to face threats like malware attacks and virus attacks during use (Hazrati et al., 2022; Khanna et al., 2024; Pastor et al., 2025). For example, a farm’s AI-powered plant management platform could be targeted by a cyberattack from a competitor, which could tamper with irrigation or fertilization instructions, leading to abnormal plant growth.
2. User Privacy Breach Risk. Data privacy and security are of great importance (Hazrati et al., 2022; Mmbando, 2025). However, highly data-dependent smart agriculture generates and records large-scale user data in real time, including user traces and sensitive crop data. Farmers lack strong awareness of network and data security. It can easily lead to data privacy breaches and information security risks (Balaska et al., 2023; Gao et al., 2024; Kumar et al., 2022).

### 3.3 Ecological risks of AI in agriculture

Consequently, human activities have environmental impacts on the ecological system (Cote and Nightingale, 2012; Dorward, 2014; Yang and Sono, 2025). From the perspective of ecological subsystems (**Table 4**), the application of AI technologies may involve an over-pursuit of efficiency gains while ignoring environmental considerations, thereby causing unintended negative impacts on ecosystems (Arevalo-Royo et al., 2025; Galaz et al., 2021; Khanna et al., 2024; Yu et al., 2024).

**Table 4:** Overview of ecological risks of AI in agriculture.

Subsystem	Risk category	Key manifestation	Representative references
Ecological	Biodiversity loss	AI-driven homogeneous planting recommendations reduce crop diversity	Leal Filho and Gbaguidi, 2024; Sajith et al., 2022; Javed et al., 2025
	Uncertainty climate risks	AI-driven expansion of agriculture and livestock increases energy consumption, carbon emissions, and deforestation	Omotayo et al., 2025; Licardo et al., 2024; Sandbrook, 2025
	Potential environmental pollution	Erroneous AI decisions may lead to the overuse of fertilizers, pesticides, and nanomaterials, resulting in soil and water pollution	Arevalo-Royo et al., 2025; Xu et al., 2022

#### 3.3.1 Biodiversity loss

In theory, AI can help sustainable plant protection and biodiversity protection (Pandey and Pandey, 2023). As AI in agriculture is currently in its exploration and implementation phase, it may have some unintended negative impacts on biodiversity (Sajith et al., 2022; Sandbrook, 2025). For instance, in the same region, AI may provide similar farmers with homogeneous planting recommendations, leading to the problem of single crop cultivation and suppressing biodiversity (Leal Filho and Gbaguidi, 2024). AI-driven automated decision-making may fail to distinguish which species in a garden should be removed, potentially eliminating beneficial plants (Javed et al., 2025). Furthermore, AI technology can precisely destroy pests, but as mentioned above, AI technology faces risks of instability and inaccuracy. The complexity of farmland can limit the effectiveness of AI-based pest management (Javed et al., 2025). It may lead to excessive pest control, thereby damaging the biodiversity of agricultural fields and plant communities, which is contrary to the principles of sustainable plant protection and agricultural production.

#### 3.3.2 Uncertainty climate risks

Large-scale deployment and application of AI in agriculture may lead to large-scale, uncontrolled expansion of agriculture and livestock, leading to the shrinkage of plant communities such as forests and grasslands, and weakening the carbon sequestration capacity of plants, associated with a reduction in forest resources, increased carbon emissions, and impacts on the global climate. Research indicates that agricultural expansion driven by technological applications can increase deforestation and environmental decline, thereby bringing climate risks (Alam et al., 2023; Omotayo et al., 2025). For example, AI may tends to promote monoculture planting, leading to the clearing of vast swathes of forest, which causes native plant species to disappear and exacerbates global warming.

In addition, AI in agriculture will also increase energy consumption, leading to indirect impacts on the climate. AI technologies such as AI and robotics are known to have high energy demands (Licardo et al., 2024; Sandbrook, 2025). The excessive energy consumption associated with AI is accompanied by a rapid growth in electricity generation. High energy consumption, associated with rapid growth in electricity generation, may be accompanied by issues such as deforestation and increased carbon emissions that impact the climate.

#### 3.3.3 Potential environmental pollution

Unlike traditional digital technologies, AI in agriculture primarily enhances efficiency by providing agricultural and planting decisions to farmers. However, as previously mentioned, the accuracy of AI decision-making can be influenced by various factors, and AI cannot guarantee absolute precision (Visser et al., 2021). This may lead AI models to generate wrong agricultural decisions, misleading farmers into adopting wrong agricultural practices, such as fertilization, irrigation, and pesticide application. The wrong decisions are not conducive to the ecological environment and plant protection, potentially causing pollution issues in the agricultural ecological system.

For instance, while AI could effectively reduce fertilizer use for agricultural producers, it sometimes generates incorrect and imprecise decisions for irrigation, fertilization, and other practices (Arevalo-Royo et al., 2025). In this context, users may adopt recommendations such as excessive fertilization and overuse of pesticides, leading to environmental pollution issues that directly harm plant health and reduce crop quality. Furthermore, in the application of AI in agriculture, various new materials, including nanomaterials, may be used (Ur Rahim et al., 2021). Although these new materials help improve efficiency, they may also cause some unintended soil pollution issues. Research indicates that nanoparticles and other “additives” used in modern, smart agriculture, may weaken soil microbial capabilities and reduce soil ecosystem functions (Xu et al., 2022), thereby threatening plant productivity and reducing agricultural output.

## 4 Mitigation strategies for systemic risks of AI in agriculture

**Figure 4:**
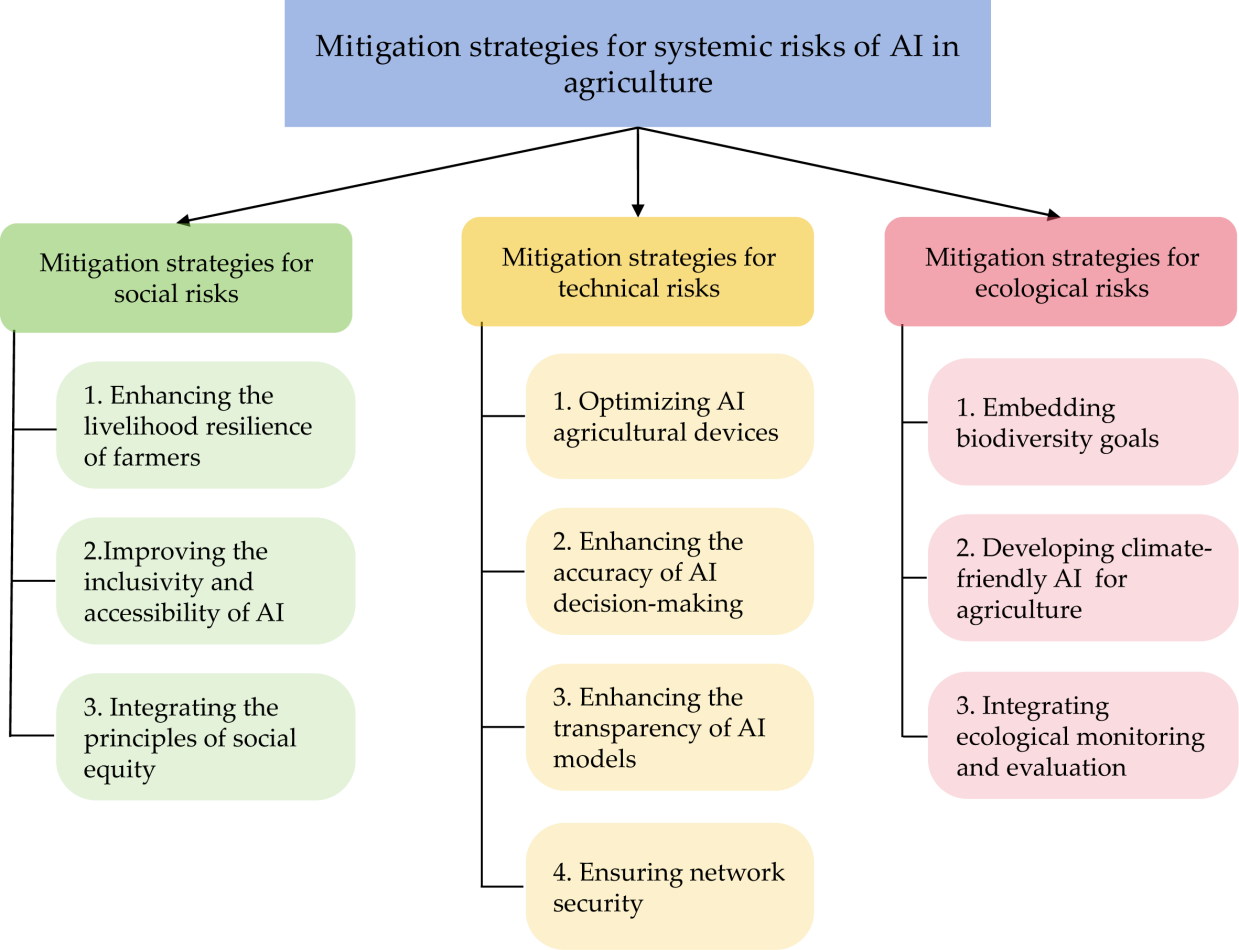
Mitigation strategies for systemic risks of AI in agriculture.

To avoid systemic risks associated with AI in agriculture, this paper suggests a comprehensive risk mitigation strategy for social, technical, and ecological risks (**Figure 4**). In the social subsystem, it is important to enhance the livelihood resilience of farmers, improve the inclusivity and accessibility of AI, and integrate the principles of social equity. From the perspective of technical subsystems, efforts should be made to optimize AI agricultural devices, enhance the accuracy of AI decision-making, enhance the transparency of AI models, and ensure network security. For the ecological subsystem, it should embed biodiversity goals, develop climate-friendly AI agricultural systems, and integrate ecological monitoring and evaluation capabilities.

### 4.1 Mitigation strategies for the risks of social subsystem

Three targeted mitigation approaches are formulated to tackle social risks and ease unemployment, inequality, and social exclusion brought by AI-driven agricultural development (**Table 5**).

**Table 5:** Mitigation strategies for the risks of social subsystem.

Subsystem	Strategy	Specific measures	Expected outcomes
Social	Enhancing livelihood resilience of farmers	Provide AI skills training; create new job types (e.g., drone pilots)	Reduces unemployment risk; helps farmers’ job transition
	Improving inclusivity and accessibility of AI	Develop low-cost AI tools; promote open-source platforms; provide digital education for smallholders	Reduces the digital divide; enables equitable access to AI
	Integrating principles of social equity	Embed fairness into data collection and algorithm design; test models across diverse farm scales and regions	Prevents algorithmic bias; ensures AI benefits all farmers

#### 4.1.1 Enhancing the livelihood resilience of farmers

Following James Scott’s moral economics framework, while using AI technology to enhance agricultural efficiency, it is also essential to ensure all farmers have the capacity for sustainable livelihoods. This approach prevents farmer unemployment and unsustainable livelihoods resulting from “AI replacing human labor”.

To do this, it is important to provide farmers with systematic AI education and skills training to enhance their ability to adapt to AI, gradually enhance farmers’ digital literacy, ensure they have opportunities to enter AI-driven agriculture, and develop new jobs (Gonzalez-Rodriguez et al., 2024; Rotz et al., 2019). For example, drones are widely used in AI-driven plant management; training selected farmers as certified drone operators could facilitate their occupational transition and create new employment opportunities within the plant protection sector.

#### 4.1.2 Improving the inclusivity and accessibility of AI

There is a need to reduce the technological gap and lower the barriers to AI adoption, enhance its accessibility and inclusivity, and promote social equality. From the AI user perspective, digital education and AI training should be provided to farmers, especially smallholder farmers with limited resources, to improve their digital literacy, reduce the digital divide among farmers, and ensure that all farmers can benefit from AI technology (Fuentes-Penailillo et al., 2024; Issahaku and Abdulai, 2020; F. Yuan et al., 2025; Zscheischler et al., 2022). From the perspective of AI developers, the goal is to design low-cost, inclusive agricultural AI technologies, promote open-source AI tools, and foster equal access for users, especially low-income smallholder farmers, to share the benefits of AI in agriculture (Leal Filho and Gbaguidi, 2024; Mmbando, 2025). For example, developing a free AI-powered mobile app that allows farmers to identify pest infestations on mainstream crops simply by taking a photo.

#### 4.1.3 Integrating the principle of social equity

It is important to establish fairness as one of the core principles of agricultural AI, overcoming potential data biases and algorithmic biases (Sandbrook, 2025). The principle of social equity should be embedded early in processes such as data collection and algorithm development. Before the model is officially put into use, it is also important to test the effect of AI models across diverse agricultural environments, regions, scales, and among different farmers. This enables the early identification and resolution of various bias issues, ensuring AI decisions do not generate biases related to scale, geography, or other factors. Such practices guide the development of AI in agriculture toward fairness (Bozeman et al., 2024; Gardezi et al., 2024). For example, before promoting and deploying AI-based plant disease identification applications, they should be tested in both plains and mountainous areas, as well as on large- and small-scale farmland, to ensure that the accuracy of plant disease identification remains consistent across these environments.

### 4.2 Mitigation strategies for the risks of technical subsystem

Four practical countermeasures are put forward to resolve technical defects, so as to elevate equipment stability, optimize decision-making precision, improve model interpretability, and reinforce network security of agricultural intelligent systems (**Table 6**).

**Table 6:** Mitigation strategies for the risks of technical subsystem.

Subsystem	Strategy	Specific measures	Expected outcomes
Technical	Optimizing AI agricultural devices	Improve device materials; regular maintenance and cleaning of technical devices; provide device maintenance and replacement services	Enhances device reliability in complex agricultural environments
	Enhancing accuracy of AI decision-making	Enrich training datasets with diverse scenario and data; improve model adaptability across different regions	Reduces errors in identification and decision-making
	Enhancing transparency of AI models	Implement explainable AI; establish decision traceability mechanisms; strengthen human-machine collaboration	Builds user trust; enables accountability for erroneous decisions
	Ensuring network security	Integrate security modules; set up intrusion testing mechanisms; strengthen privacy protection	Protects against cyberattacks and data breaches

#### 4.2.1 Optimizing AI agricultural devices

AI agricultural devices are constantly placed in humid, sunny, and other agricultural environments that are not suitable for electronic devices. It is necessary to optimize AI agricultural devices to enhance their resilience. First, it is suggested to explore improvements in the structural materials of technical devices deployed in agricultural environments, such as introducing nanostructured functional materials that mitigate environmental impacts, including humidity and corrosion, on device performance, thereby enhancing their resilience and operational longevity (Dai et al., 2023). Second, regular maintenance and cleaning of technical devices should be conducted to prevent damage to various agricultural equipment caused by external environmental factors (Lakhiar et al., 2025). Third, to provide comprehensive device maintenance and replacement services. Particularly during peak farming seasons, agricultural device instability can severely disrupt agricultural production processes, putting farmers at risk. Technology providers must offer timely device repair or replacement services to promptly deal with malfunctioning devices, ensuring the stable running of AI in agriculture. For example, regularly cleaning the sensors on plant protection equipment, promptly updating and calibrating AI-based plant disease recognition models, thereby promoting the sustainable development of AI-driven plant protection.

#### 4.2.2 Enhancing the accuracy of AI decision-making

1. Enriching Agricultural AI Training Datasets. The accuracy of AI models highly depends on data, which is also the key factor determining AI competitiveness. The more data used for training, the stronger the AI performance becomes. Therefore, it is essential to collect sufficient crop data and agricultural production data to enhance both the quantity and quality of training data for AI agricultural models (Cui et al., 2024; Schmidt et al., 2022; Warner et al., 2020). For instance, from the perspective of data collection, agricultural production data related to plants, such as plant growth indicators and crop health status, can be comprehensively gathered across different areas, seasons, weather, and other factors, so as to cover diverse planting scenarios as much as possible, enriching the training dataset for AI agricultural models (Chen et al., 2021; Doutoum and Tugrul, 2025; Yang et al., 2025). From the perspective of data sharing, it is important to promote agricultural data, such as on plant health, crop growth, and pest incidence, exchange among different regions, farmer groups, and agricultural databases. Based on this, strict data security measures shall be established to improve the completeness of training data (Buyuktepe et al., 2025; Johnson et al., 2024; Tawfeek et al., 2023).
2. Enhancing AI adaptability across diverse agricultural scenarios. As previously noted, current general-purpose AI models often fail when applied to different agricultural contexts. When applying specific AI models to different agricultural contexts, it is necessary to optimize and refine the models based on local natural conditions and social infrastructure (Qi et al., 2025). Specifically, it is necessary to keep optimizing algorithm design, develop advanced algorithms that can adapt to diverse agricultural situations, test and adjust them in different geographical areas, seasons, and weather conditions, ensure the AI model has greater adaptability (Anjum et al., 2025; Mmbando, 2025; Shoaib et al., 2022; Su et al., 2023). For example, algorithms for identifying plant pests and diseases should undergo cross-regional validation, to ensure they are more adaptable.

#### 4.2.3 Enhancing the transparency of AI models

It is necessary to enhance the transparency of model operations, so as to overcome the black-box nature of AI technology. This enables non-professional users to gain a basic understanding of the working mechanisms of agricultural models, machine learning, and deep learning technologies, and to comprehend the logic behind how models generate specific decisions (Gardezi et al., 2024; Izquierdo-Bueno et al., 2024; Oikonomidis et al., 2023). Specifically, on the one hand, it can try to optimize the model system, translating specialized and complex decision-making solutions into understandable, actionable, and highly intuitive outcomes for farmers, thereby avoiding the risk of non-traceable responsibility (Kpodo and Nejadhashemi, 2025). At the same time, a decision traceability mechanism should be established to record key data through the decision-making process. For example, when AI models recommend plant protection products, the system should record and store images of plant diseases, probability assessments, and weather conditions to facilitate accountability in the future.

On the other hand, it should strengthen human-machine collaboration mechanisms, clarify AI’s supporting role rather than its dominant position in agricultural decision-making, integrate AI recommendations with farmers’ experience, so as to build more understandable and transparent algorithmic systems (Licardo et al., 2024; Stodle et al., 2025). For example, AI can identify crop diseases and recommend pesticide application strategies, but the final decision on whether to apply pesticides rests with the farmers themselves.

#### 4.2.4 Ensuring network security

It is necessary to pay more attention to network security risks associated with AI-driven agriculture, and adopt various defensive measures. Specifically, it could involve integrating security measures modules into deep learning models, setting up intrusion testing mechanisms in AI systems, and adding junk mail filtering functions (Maraveas et al., 2023; Yuan et al., 2025; Zogaan et al., 2025). At the same time, increase campaign activities in rural communities about the risks of network attacks (Sparrow et al., 2021), helping AI users recognize various ways of network attacks. Additionally, privacy protection of AI in agriculture would be strengthened (Ding et al., 2023), in order to ensure farmers do not face privacy risks arising from external attacks or internal breaches. For example, farmers can be taught not to click on text messages they receive on their phones regarding crop subsidies or pesticide distribution, thereby raising their security awareness.

### 4.3 Mitigation strategies for the risks of ecological subsystem

Three effective regulatory and developmental solutions are constructed to offset ecological adverse effects, curbing biodiversity loss, climate hazards, and environmental pollution induced by the popularization of agricultural AI technologies (**Table 7**).

**Table 7:** Mitigation strategies for the risks of ecological subsystem.

Subsystem	Strategy	Specific measures	Expected outcomes
Ecological	Embedding biodiversity goals	Encourage diverse planting recommendations; provide ecological subsidies for diversified farming	Maintains ecological balance; reduces the risk of pest outbreaks and the spread of crop diseases
	Developing climate-friendly AI for agriculture	Design energy-efficient AI systems; strengthen scientific planning to avoid uncontrolled agricultural expansion	Reduces carbon emissions and energy consumption
	Integrating ecological monitoring and evaluation	Combine AI with drones and sensor for real-time ecological monitoring; evaluate environmental impacts of AI decisions	Enables early detection and mitigation of unintended ecological damage

#### 4.3.1 Embedding biodiversity goals

In the design and application process of AI-driven agriculture, it is necessary to embed the goal of biodiversity, so that the AI model can make precise decisions while taking diversity into account (Ntawuruhunga et al., 2023; Pandey and Pandey, 2023). For example, AI models should take into account the crop types and growing conditions of different farmers, providing diverse plant protection recommendations, while also promptly issuing warnings about the risk of pesticide resistance that may arise from long-term reliance on a single pesticide. Furthermore, as complex AI models embedded with biodiversity objectives may increase user costs, policymakers should provide farmers with specific ecological benefits, so as to encourage and support their adoption of AI models with biodiversity goals (Awiti et al., 2022). For example, provide additional subsidies to farmers who have diversified their crops.

#### 4.3.2 Developing climate-friendly AI for agriculture

To solve the potential negative impacts of AI in agriculture on the climate, there is a need to increase investment in climate-smart AI agricultural solutions (Omotayo et al., 2025). On the one hand, it is necessary to design energy-efficient AI systems in order to reduce energy consumption and lower carbon emissions. For example, designing a low-power, solar-powered AI device for pest and disease identification, it can monitor crop growth without increasing carbon emissions. On the other hand, the scientific planning of AI in agriculture should be strengthened, so as to avoid potential uncontrolled expansion of agriculture, and prevent excessive deforestation. Furthermore, collaboration between AI experts and climate scientists should be strengthened. This will help them jointly develop climate-friendly AI agricultural systems, balance the advancement of AI technology with climate adaptation goals (Essenfelder et al., 2025).

#### 4.3.3 Integrating ecological monitoring and evaluation

It is essential to fully consider the potential environmental pollution issues arising from AI in agriculture. This requires integrating ecological monitoring and evaluating functions to enhance AI’s sensitivity to ecological changes, enabling the timely identification of unintended environmental problems (Husaini and Sohail, 2024; Yang et al., 2025).

On the one hand, integrating agricultural ecological monitoring functions and processes. It can be attempted to combine AI models with drones, robots, and early warning systems to conduct dynamic agricultural ecological monitoring. This approach allows for the real-time transmission and analysis of data from agricultural lands, enabling prompt identification of various abnormal ecological changes, so as to reduce potential negative environmental impacts (Salybekova et al., 2024; Shehu et al., 2025). On the other hand, integrating agricultural ecological evaluation functions and procedures. This requires not only evaluating the environmental nature of new materials related to AI in agriculture, but also evaluating the potential negative ecological impacts of decisions made by specific AI models, so as to mitigate and correct potential ecological issues (Badshah et al., 2024; McClements et al., 2021). For example, before introducing new types of nano-pesticides, it is necessary to assess their long-term effects on the soil and crop root systems in advance. Similarly, if an AI model frequently recommends a specific insecticide, the long-term effects of that insecticide on plants must be monitored.

## 5 Discussion

### 5.1 Potential systemic cross-risks

Based on literature review and in-depth reflection, we found that the true crisis of AI-driven agriculture is not the risks of individual subsystems, but rather the mutual interaction and expansion of risks across the social, technical, and ecological subsystems. This leads to an imbalance in the STES, forming complex multiple risks that bring negative effects to agricultural production and plant protection. We attempt simple reasoning and deduction:

1. If we focus only on the technical subsystem, emphasizing the widespread adoption of AI technologies in rural communities and among farmers, it may bring risks to the social system and the ecological system. For instance, farmers lacking digital literacy and unable to afford AI costs may lose their agricultural livelihood opportunities, before they even have a chance to participate in technology skills training (Yang and Zheng, 2026). Research has shown that AI can significantly enhance agricultural efficiency and planting management; however, if widely adopted, it would primarily benefit only a small number of farmers managing large-scale farmland and major crops, potentially excluding minor crops and smallholders (Gardezi et al., 2024). Furthermore, the rapid adoption and widespread expansion of AI in agriculture can significantly increase the deployment of various intelligent hardware devices, compared to natural planting and traditional agricultural production. These large-scale modern facilities consume significant computing power and energy (Cai et al., 2025), thereby increasing rural energy consumption and carbon emissions in a short time and raising ecological management costs.
2. If we focus only on the social subsystem, especially emphasizing equality, limiting the rapid development and adoption of AI to achieve fairness goals, it may hinder these regions’ integration into the modernization process. For instance, in rural areas dominated by smallholders, only a minority of farmers possessing sufficient resources and digital literacy have the capacity to use AI. In this context, a strategy of not introducing AI could be adopted if the sole objective is social equality. However, in the era of intelligence, if the intelligent transformation of agriculture is not progressively advanced, it will prevent agricultural production from achieving more precise management and hinder its integration into modern agricultural supply chains (Aylak, 2021; Hu et al., 2024), and will also threaten plant protection. Meanwhile, if AI applications are widely restricted in pursuit of absolute fairness, many sectors will continue to adopt traditional planting practices. Without precise agrochemical application, over-fertilization will occur, which in turn triggers ecological risks and threatens plant diversity.
3. If we focus only on the ecological subsystem, setting extremely stringent agricultural ecological standards, this will also bring risks for other subsystems. On the one hand, the extremely stringent agricultural ecological standards entail complex review procedures and certification processes. This not only increases costs, but may also lead to bureaucratic certification issues, bringing challenges for farmers (Barrett et al., 2001). On the other hand, some studies indicate that increasing investment in agricultural research and development can significantly reduce emissions (Alkara et al., 2026). Facing stringent ecological standards, this also means that AI enterprises must increase their R&D investments, allocating more funds to the design of eco-friendly materials, energy-saving algorithms, and similar innovations. These efforts will inevitably increase the costs of AI-driven agriculture, thereby increasing expenses for users, increasing the gap in AI application among farmers with different socio-economic conditions. At the same time, to achieve stringent ecological standards, companies’ R&D cycles have extended, which may also limit AI’s practical application in the field and limit innovation of AI in agriculture.

Therefore, AI in agriculture not only faces risks from individual subsystems, but also brings various complex, intertwined risks that arise from the interplay among these subsystems. The absence of rules is an important cause of systemic cross-risk of AI in agriculture (Nayal et al., 2023). From the perspective of each subsystem: the technical subsystem focuses on enhancing efficiency, the social subsystem emphasizes fairness and justice, and the ecological subsystem concerns ecological protection. In the absence of common rules, the objectives of the three subsystems may easily differ and conflict. In the process of the three subsystems interacting with one another, any action taken to achieve the objectives of one subsystem may cause risks in the other subsystems, ultimately leading to an unbalanced agricultural system as a whole. This could lead to some issues, such as unclear responsibilities and confusing standards, thereby limiting the sustainable development of AI in agriculture (Pastor et al., 2025; Uyeh et al., 2023).

### 5.2 Mitigation strategies for STES: a collaborative governance framework for AI in agriculture

**Figure 5:**
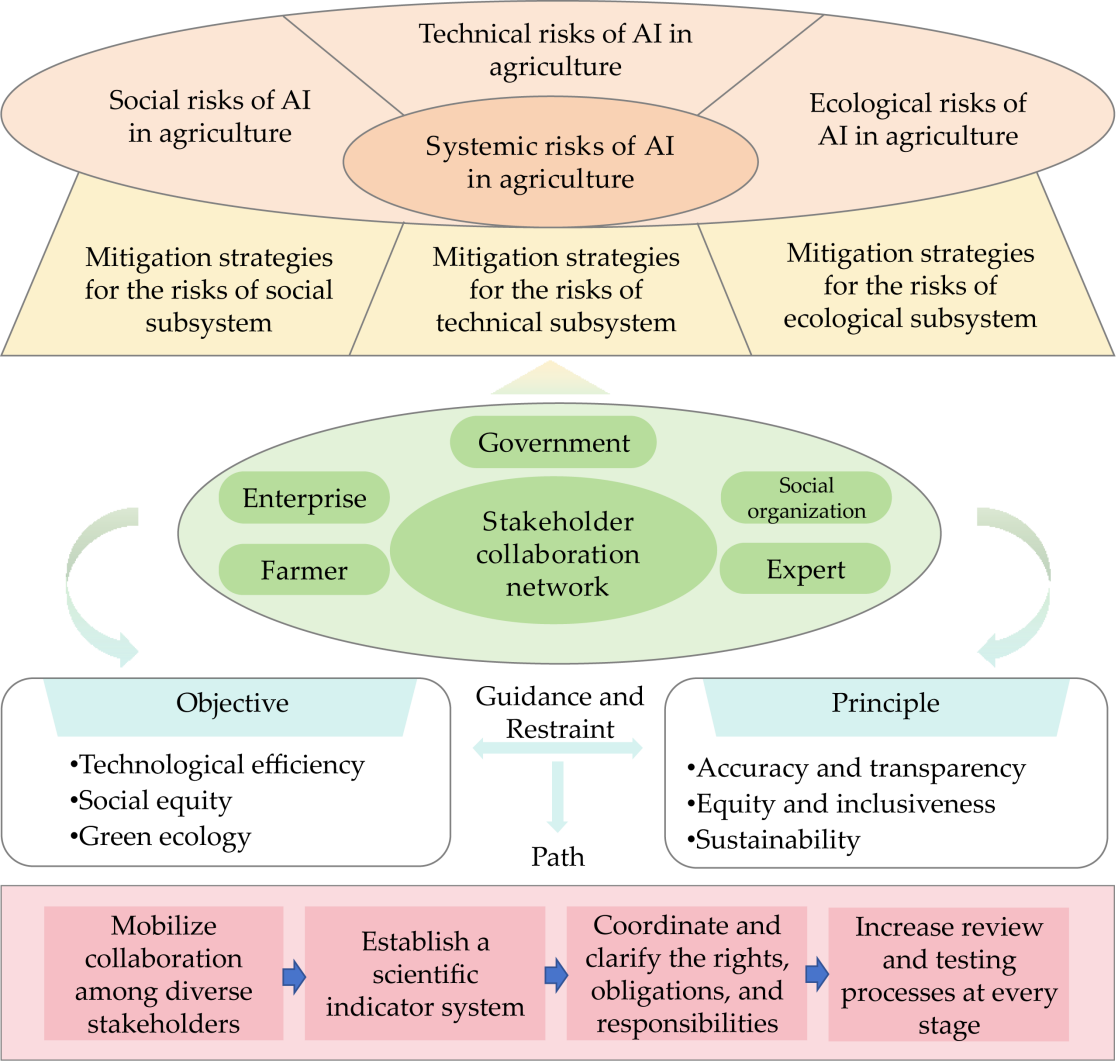
Collaborative governance framework for AI in agriculture.

From the Social-Technical-Ecological System (STES) perspective, to effectively mitigate systemic risks from AI in agriculture, it is necessary to establish a collaborative governance framework that facilitates the participation of diverse stakeholders, including governments, farmers, enterprises, social organizations, and experts (**Figure 5**), so as to achieve the goals of technical efficiency, social equity, and green ecological (Cesco et al., 2024; Chen et al., 2025; Natarajan and Chandrasekaran, 2025). This framework centers on three core objectives: enhancing technical efficiency, ensuring social equity, and promoting ecological sustainability, while adhering to three guiding principles: accuracy and transparency, equity and inclusiveness, and long-term sustainability. Building upon these foundations, we propose a clear and actionable governance pathway.

First, establish a multi-stakeholder governance entity for AI-driven agriculture, including government, farmers, AI providers, social organizations, sociologists, and ecologists. Second, it is necessary to establish normalized development standards and scientific indicators for AI-driven agriculture. These standards should be based on the diverse interests of multiple stakeholders and actively incorporate relevant research findings, including this paper (Sun et al., 2024; Arevalo-Royo et al., 2025; Sparrow et al., 2021). Specifically, we propose developing an indicator system that promotes STES equilibrium, thereby quantifying the implementation pathway for AI in agriculture. This system encompasses a social equity index, biodiversity standard coefficients, and other relevant metrics. On this basis, local government should introduces operational guidelines and supporting policies to safeguard the interests of all stakeholders (Ali et al., 2024; Balaska et al., 2023). Third, a forward-looking governance system for AI in agriculture must be established. Before AI technology is used for specific local agricultural development, it is necessary to coordinate and clarify the rights, obligations, and responsibilities of all participating stakeholders (Arevalo-Royo et al., 2025; Pimenow et al., 2025; Regan, 2019), so as to ensure that each step balances technical efficiency, green ecological sustainability, and social equity.

Finally, in summary, we reflectively construct a risk governance framework for AI in agriculture: 1) Mobilize collaboration among diverse stakeholders, including government, farmers, experts, and social organizations; 2) Establish a scientific indicator system, including the AI accuracy rate, social equality index, and ecological environmental protection index; 3) Coordinate and clarify the rights, obligations, and responsibilities among participants through meetings and discussions; 4) Increase review and testing processes at every stage, identify potential risks as early as possible, and minimize them to the greatest extent. This approach will reduce the risks and promote the sustainable development of AI in agriculture.

### 5.3 Limitations and future prospects

Of course, as AI in agriculture remains in an ongoing exploration phase, and practices have varied significantly across different regions, this study still has certain limitations. 1) Some studies indicate that policy differences across countries and regions can impact AI in agriculture (Omotayo et al., 2025), and the effectiveness and risks of agricultural AI vary significantly across different regions (Arevalo-Royo et al., 2025). Our study did not fully explore risk differences across various contexts, such as those between developed and developing countries, or between smallholders and corporate farms. This limitation to some extent restricts the applicability of the research conclusions. 2) This review is limited to the Web of Science and Scopus databases, and is only available in English publications. This might ignore valuable insights from non-English sources, potentially limiting global comprehensiveness. 3) The screening and organization of literature mainly rely on manual reading and evaluation by the research team, without incorporating bibliometric tools such as citation network analysis or co-occurrence mapping. Although this method is in-depth, it may not fully capture broader quantitative patterns, emerging trends, or interconnections in the field. 4) This study adopts the STES framework for analysis of the risks of AI in agriculture, primarily to consider risks such as algorithmic inaccuracy, social inequity, negative ecological impacts, and so on, from technical, social, and ecological dimensions. It does not comprehensively cover all other potential risk forms, lacking targeted analyses around political, economic, cultural, and other dimensions.

Based on this, in the future, it should be improved and refined in the following directions, so as to further enrich the research findings: Future research may select different countries and regions for comparative case studies, systematically examining the differences in risks associated with AI in agriculture across countries and regions at varying levels of development, as well as under different agricultural production models and technological conditions. This approach will enable the development of more targeted risk mitigation strategies. At the same time, it should expand the databases such as CNKI and Google Scholar, select both English and non-English literatures, and consider incorporating various appropriate bibliometric analysis methods (Reza et al., 2025). And it would also be beneficial to combine qualitative and quantitative methods to establish a transparent risk assessment framework. Building on this foundation, it will actively explore the potential deep impact of AI-driven agriculture across economic, political, and cultural dimensions, so as to enrich relevant research results. Looking forward to the future, the government should allocate more resources to support the application of AI technologies in plant protection and agricultural production, fully consider diverse development goals, including technical efficiency, social equity, and green ecology, commit to establish a responsible, people-centered governance framework for AI in agriculture (Leal Filho and Gbaguidi, 2024; McClements et al., 2021; Tzachor et al., 2022), in order to advance plant protection, global food security, and sustainable agricultural development.

## 6 Conclusion

This study adopts a narrative review methodology, from the STES analytical framework, to conduct a comprehensive analysis of the risks from AI in agriculture. The research finds that AI in agriculture faces a multitude of risks across the social, technical, and ecological subsystems, including: social unemployment, social inequity, systemic exclusion, device uncertainty, decision-making failure, the black-box nature of AI, network security threats, suppression biodiversity, potential climate risks, agricultural environmental pollution, and so on. Moreover, the lack of common rules and a well-developed multi-stakeholder governance mechanism for AI in agriculture may lead to cross risks, and cause imbalances in the agricultural system, thereby posing a threat to the sustainability of plant protection and agricultural development.

This study presents a series of mitigation strategies: optimizing agricultural technical devices, enhancing the precision and understandability of AI decisions, ensuring network security, strengthening the livelihood resilience of farmers, promoting AI inclusiveness, integrating the principles of social equity, embedding biodiversity goals and plant protection, developing climate-friendly AI for agriculture, integrating ecological monitoring and evaluation functions, and other strategies. The overall goal is to balance systemic goals of AI in agriculture, such as technical efficiency, social equity, and green ecological. This requires establishing a systematic governance framework for AI in agriculture among diverse stakeholders, commits to achieving technical efficiency, social equity, and green ecological goals.

It should be noted that this study’s identification of risks in AI-driven agriculture does not deny the potential of AI in agriculture, nor does it incite panic regarding these risks. Rather, it seeks to encourage stakeholders to balance the risks and benefits of AI, to fully recognize its double-edged sword effect in agriculture, and to avoid falling into a technological deterministic perspective. Finally, although this study is framed within the broader context of agriculture, as plants are at the core of agricultural systems, the risks identified in this paper from social, technical, and ecological perspectives can help promote the responsible application of smart technologies in plant protection and development, providing theoretical support and practical insights for enhancing agricultural productivity and fostering green ecological outcomes.

## References

[B1] Ahlborg H. Ruiz-Mercado I. Molander S. Masera O. (2019). Bringing technology into social-ecological systems researchmotivations for a socio-technical-ecological systems approach. Sustainability 11, 2009. doi: 10.3390/su11072009. PMID: 30654563

[B2] Alam M. F. B. Tushar S. R. Zaman S. M. Gonzalez E. D. R. S. Bari A. B. M. M. Karmaker C. L. (2023). Analysis of the drivers of agriculture 4.0 implementation in the emerging economies: Implications towards sustainability and food security. Green Technol. Sustainability 1, 100021. doi: 10.1016/j.grets.2023.100021. PMID: 38826717

[B3] Ali F. Razzaq A. Tariq W. Hameed A. Rehman A. Razzaq K. . (2024). Spectral intelligence: AI-driven hyperspectral imaging for agricultural and ecosystem applications. Agronomy-Basel 14, 2260. doi: 10.3390/agronomy14102260. PMID: 30654563

[B4] Alkara I. Urak F. Turkoglu D. Bilgic A. Dogan M. (2026). From soil to sustainability: The role of agricultural R&D in decarbonization. Sustain. Dev 1–25. doi: 10.1002/sd.70771. PMID: 41531421

[B5] Anandhakrishnan T. Jaisakthi S. M. (2022). Deep convolutional neural networks for image based tomato leaf disease detection. Sustain. Chem. Pharm. 30, 100793. doi: 10.1016/j.scp.2022.100793. PMID: 38826717

[B6] Anjum M. Kraiem N. Min H. Dutta A. K. Daradkeh Y. I. Shahab S. (2025). Big data-driven agriculture: A novel framework for resource management and sustainability. Cogent Food. Agric. 11, 2470249. doi: 10.1080/23311932.2025.2470249. PMID: 37339054

[B7] Arevalo-Royo J. Flor-Montalvo F.-J. Latorre-Biel J.-I. Jimenez-Macias E. Martinez-Camara E. Blanco-Fernandez J. (2025). AI guidelines for sustainable rural development and climate resilience in resource-constrained regions. Sustain. Dev 33, 9385–9397. doi: 10.1002/sd.70160. PMID: 41531421

[B8] Awiti H. A. Gido E. O. Obare G. A. (2022). Smallholder farmers climate-smart crop diversification cost structure: Empirical evidence from western Kenya. Front. Sustain. Food Syst. 6, 842987. doi: 10.3389/fsufs.2022.842987

[B9] Aylak B. L. (2021). Artificial intelligence and machine learning applications in agricultural supply chain: A critical commentary. Fresenius Environ. Bull. 30, 8905–8916. Available online at: https://webofscience.clarivate.cn/wos/woscc/full-record/WOS:000678352300010.

[B10] Badshah A. Alkazemi B. Y. Din F. Zamli K. Z. Haris M. (2024). Crop classification and yield prediction using robust machine learning models for agricultural sustainability. IEEE Access 12, 162799–162813. doi: 10.1109/ACCESS.2024.3486653. PMID: 25079929

[B11] Balaska V. Adamidou Z. Vryzas Z. Gasteratos A. (2023). Sustainable crop protection via robotics and artificial intelligence solutions. Machines 11, 774. doi: 10.3390/machines11080774. PMID: 30654563

[B12] Barbosa A. Hovakimyan N. Martin N. F. (2020). Risk-averse optimization of crop inputs using a deep ensemble of convolutional neural networks. Comput. Electron. Agric. 178, 105785. doi: 10.1016/j.compag.2020.105785. PMID: 38826717

[B13] Barrett H. R. Browne A. W. Harris P. J. C. Cadoret K. (2001). Smallholder farmers and organic certification: Accessing the EU market from the developing world. Biol. Agric. Horticulture 19, 183–199. doi: 10.1080/01448765.2001.9754920. PMID: 37339054

[B14] Beck U. (2011). Cosmopolitanism as imagined communities of global risk. Am. Behav. Scientist 55, 1346–1361. doi: 10.1177/0002764211409739

[B15] Bowden C. Foster T. Parkes B. (2025). Risk of rice production failure in India under climate change. Environ. Res. Lett. 20, 094038. doi: 10.1088/1748-9326/adf459

[B16] Bozeman J. F. Hollauer C. Ramshankar A. T. Nakkasunchi S. Jambeck J. Hicks A. . (2024). Embed systemic equity throughout industrial ecology applications: How to address machine learning unfairness and bias. J. Ind. Ecol. 28, 1362–1376. doi: 10.1111/jiec.13509. PMID: 39722860 PMC11667658

[B17] Bui H. T. Aboutorab H. Mahboubi A. Gao Y. Sultan N. H. Chauhan A. . (2024). Agriculture 4.0 and beyond: Evaluating cyber threat intelligence sources and techniques in smart farming ecosystems. Comput. Secur. 140, 103754. doi: 10.1016/j.cose.2024.103754. PMID: 38826717

[B18] Buyuktepe O. Catal C. Kar G. Bouzembrak Y. Marvin H. Gavai A. (2025). Food fraud detection using explainable artificial intelligence. Expert Syst. 42 (1), e13387. doi: 10.1111/exsy.13387. PMID: 40046247

[B19] Cai W. Bu K. Zha L. Zhang J. Lai D. Bao H. (2025). Energy consumption of plant factory with artificial light: Challenges and opportunities. Renewable Sustain. Energy Rev. 210, 115235. doi: 10.1016/j.rser.2024.115235. PMID: 38826717

[B20] Cesco S. Ascoli D. Bailoni L. Bischetti G. B. Buzzini P. Cairoli M. . (2024). Smart management of emergencies in the agricultural, forestry, and animal production domain: Tackling evolving risks in the climate change era. Int. J. Disaster Risk Reduct. 114, 105015. doi: 10.1016/j.ijdrr.2024.105015. PMID: 38826717

[B22] Chen M. Cui Y. Wang X. Xie H. Liu F. Luo T. . (2021). A reinforcement learning approach to irrigation decision-making for rice using weather forecasts. Agric. Water Manage. 250, 106838. doi: 10.1016/j.agwat.2021.106838. PMID: 38826717

[B21] Chen H. Gao B. Li Y. (2025). Soil pollution and remediation: Emerging challenges and innovations. Front. Environ. Sci. 13, 1606054. doi: 10.3389/fenvs.2025.1606054

[B23] Cote M. Nightingale A. J. (2012). Resilience thinking meets social theory: Situating social change in socio-ecological systems (SES) research. Prog. Hum. Geogr. 36, 475–489. doi: 10.1177/0309132511425708

[B24] Cui J.-L. Li H. He Q. Jin B.-Y. Liu Z. Zhang X.-M. . (2024). Integrating classic AI and agriculture: A novel model for predicting insecticide-likeness to enhance efficiency in insecticide development. Comput. Biol. Chem. 112, 108113. doi: 10.1016/j.compbiolchem.2024.108113. PMID: 38851150

[B25] Dai S. Li X. Jiang C. Ping J. Ying Y. (2023). Triboelectric nanogenerators for smart agriculture. Infomat 5 (2), e12391. doi: 10.1002/inf2.12391. PMID: 41531421

[B26] Denholm P. (2006). Improving the technical, environmental and social performance of wind energy systems using biomass-based energy storage. Renewable Energy 31, 1355–1370. doi: 10.1016/j.renene.2005.07.001. PMID: 38826717

[B27] Ding H. Tian J. Yu W. Wilson D. I. Young B. R. Cui X. . (2023). The application of artificial intelligence and big data in the food industry. Foods 12, 4511. doi: 10.3390/foods12244511. PMID: 38137314 PMC10742996

[B28] Dixit K. Aashish K. Dwivedi A. K. (2023). Antecedents of smart farming adoption to mitigate the digital divide—Extended innovation diffusion model. Technol. Soc. 75, 102348. doi: 10.1016/j.techsoc.2023.102348. PMID: 38826717

[B29] Dorigo T. Brown G. D. Casonato C. Cerda A. Ciarrochi J. da Lio M. . (2025). Artificial intelligence in science and society: The vision of USERN. IEEE Access 13, 15993–16054. doi: 10.1109/ACCESS.2025.3529357. PMID: 25079929

[B30] Dorward A. R. (2014). Livelisystems: A conceptual framework integrating social, ecosystem, development, and evolutionary theory. Ecol. Soc 19, 44. doi: 10.5751/ES-06494-190244

[B31] Doutoum A. S. Tugrul B. (2025). A systematic review of deep learning techniques for apple leaf diseases classification and detection. PeerJ Comput. Sci. 11, e2655. doi: 10.7717/peerj-cs.2655. PMID: 40062248 PMC11888844

[B32] Essenfelder A. H. Toreti A. Seguini L. (2025). Expert-driven explainable artificial intelligence models can detect multiple climate hazards relevant for agriculture. Commun. Earth Environ. 6, 207. doi: 10.1038/s43247-024-01987-3. PMID: 37880705

[B33] Fuentes-Penailillo F. Gutter K. Vega R. Silva G. C. (2024). New generation sustainable technologies for soilless vegetable production. Horticulturae 10, 49. doi: 10.3390/horticulturae10010049. PMID: 30654563

[B34] Galaz V. Centeno M. A. Callahan P. W. Causevic A. Patterson T. Brass I. . (2021). Artificial intelligence, systemic risks, and sustainability. Technol. Soc. 67, 101741. doi: 10.1016/j.techsoc.2021.101741. PMID: 38826717

[B35] Gao Y. Camtepe S. A. Sultan N. H. Bui H. T. Mahboubi A. Aboutorab H. . (2024). Security threats to agricultural artificial intelligence: Position and perspective. Comput. Electron. Agric. 227, 109557. doi: 10.1016/j.compag.2024.109557. PMID: 38826717

[B36] Gardezi M. Joshi B. Rizzo D. M. Ryan M. Prutzer E. Brugler S. . (2024). Artificial intelligence in farming: Challenges and opportunities for building trust. Agron. J. 116, 1217–1228. doi: 10.1002/agj2.21353. PMID: 41531421

[B37] Gautron R. Maillard O.-A. Preux P. Corbeels M. Sabbadin R. (2022). Reinforcement learning for crop management support: Review, prospects and challenges. Comput. Electron. Agric. 200, 107182. doi: 10.1016/j.compag.2022.107182. PMID: 38826717

[B38] Gonzalez-Rodriguez V. E. Izquierdo-Bueno I. Cantoral J. M. Carbu M. Garrido C. (2024). Artificial intelligence: A promising tool for application in phytopathology. Horticulturae 10, 197. doi: 10.3390/horticulturae10030197. PMID: 30654563

[B39] Goyal R. Nath A. Niranjan U. Sharda R. (2025). Analyzing the performance of deep convolutional neural network models for weed identification in potato fields. Crop Prot. 188, 107035. doi: 10.1016/j.cropro.2024.107035. PMID: 38826717

[B40] Gupta S. Tripathi A. K. (2024). Fruit and vegetable disease detection and classification: Recent trends, challenges, and future opportunities. Eng. Appl. Artif. Intell. 133, 108260. doi: 10.1016/j.engappai.2024.108260. PMID: 38826717

[B41] Han R. Liang X. Shu L. Jing X. Yang F. Tian R. (2025a). TeaWeeding-Action: A vision-based dataset for weeding behavior recognition in tea plantations. Front. Plant Sci. 16, 1722007. doi: 10.3389/fpls.2025.1722007. PMID: 41477254 PMC12748151

[B42] Han R. Zheng Y. Tian R. Shu L. Jing X. Yang F. (2025b). An image dataset for analyzing tea picking behavior in tea plantations. Front. Plant Sci. 15, 1473558. doi: 10.3389/fpls.2024.1473558. PMID: 39881732 PMC11776433

[B43] Hazrati M. Dara R. Kaur J. (2022). On-farm data security: Practical recommendations for securing farm data. Front. Sustain. Food Syst. 6, 884187. doi: 10.3389/fsufs.2022.884187

[B44] Hu Y. Tang J. Yang J. (2024). Introducing artificial intelligence technology to plant disease management for sustainable agriculture. Crop Prot. 184, 106764. doi: 10.1016/j.cropro.2024.106764. PMID: 38826717

[B45] Hughes N. Soh W. Y. Lawson K. Lu M. (2022). Improving the performance of micro-simulation models with machine learning: The case of Australian farms. Economic Model. 115, 105957. doi: 10.1016/j.econmod.2022.105957. PMID: 38826717

[B46] Husaini A. M. Sohail M. (2024). Agrochemical-free genetically modified and genome-edited crops: Towards achieving the United Nations sustainable development goals and a “greener” green revolution. J. Biotechnol. 389, 68–77. doi: 10.1016/j.jbiotec.2024.04.015. PMID: 38663518

[B47] Ibrahim I. A. Truby J. M. (2023). FarmTech: Regulating the use of digital technologies in the agricultural sector. Food Energy Secur. 12 (4), e483. doi: 10.1002/fes3.483. PMID: 41531421

[B48] Issahaku G. Abdulai A. (2020). Adoption of climate-smart practices and its impact on farm performance and risk exposure among smallholder farmers in Ghana. Aust. J. Agric. Resour. Econ. 64, 396–420. doi: 10.1111/1467-8489.12357. PMID: 40046247

[B49] Izquierdo-Bueno I. Moraga J. Cantoral J. M. Carbu M. Garrido C. Gonzalez-Rodriguez V. E. (2024). Smart viniculture: Applying artificial intelligence for improved winemaking and risk management. Appl. Sci.-Basel 14, 10277. doi: 10.3390/app142210277. PMID: 30654563

[B51] Javed Q. Bouhadi M. Ban S. G. Ban D. Heath D. Iqbal B. . (2025). Smart chip technology for the control and management of invasive plant species: A review. Plants-Basel 14, 1510. doi: 10.3390/plants14101510. PMID: 40431075 PMC12114904

[B50] Javed K. Smagghe G. Kang Y.-Q. Wang Q. Wang Y. (2025). Artificial intelligence in the mass production of natural enemies for biological control in modern agriculture. Pest. Manage. Sci 81, 7577–7592. doi: 10.1002/ps.70116. PMID: 40814223 PMC12618917

[B52] Johnson I. Mary X. A. Winifred Raj A. P. Chalmers J. Karthikeyan M. Andrew J. (2024). Deep-millet: A deep learning model for pearl millet disease identification to envisage precision agriculture. Environ. Res. Commun. 6, 105031. doi: 10.1088/2515-7620/ad8415

[B53] Kalimuthu T. Kalpana P. Kuppusamy S. Sreedharan V. R. (2024). Intelligent decision-making framework for agriculture supply chain in emerging economies: Research opportunities and challenges. Comput. Electron. Agric. 219, 108766. doi: 10.1016/j.compag.2024.108766. PMID: 38826717

[B54] Kazancoglu Y. Lafci C. Kumar A. Luthra S. Garza-Reyes J. A. Berberoglu Y. (2024). The role of agri-food 4.0 in climate-smart farming for controlling climate change-related risks: A business perspective analysis. Business Strategy Environ. 33, 2788–2802. doi: 10.1002/bse.3629. PMID: 41531421

[B55] Khanna M. Atallah S. S. Heckelei T. Wu L. Storm H. (2024). Economics of the adoption of artificial intelligence-based digital technologies in agriculture. Annu. Rev. Resource Econ 16, 41–61. doi: 10.1146/annurev-resource-101623-092515. PMID: 41139587

[B56] Kikon A. Deka P. C. (2022). Artificial intelligence application in drought assessment, monitoring and forecasting: A review. Stochastic Environ. Res. Risk Assess. 36, 1197–1214. doi: 10.1007/s00477-021-02129-3. PMID: 30311153

[B57] Klerkx L. Jakku E. Labarthe P. (2019). A review of social science on digital agriculture, smart farming and agriculture 4.0: New contributions and a future research agenda. NJAS Wageningen J. Life Sci. 90–91, 100315. doi: 10.1016/j.njas.2019.100315. PMID: 38826717

[B58] Kpodo J. Nejadhashemi A. P. (2025). Navigating challenges/opportunities in developing smart agricultural extension platforms: Multi-media data mining techniques. Artif. Intell. Agric. 15, 426–448. doi: 10.1016/j.aiia.2025.04.001. PMID: 38826717

[B63] Kumar Y. Dubey A. K. Arora R. R. Rocha A. (2022). Multiclass classification of nutrients deficiency of apple using deep neural network. Neural Comput. Appl. 34, 8411–8422. doi: 10.1007/s00521-020-05310-x. PMID: 30311153

[B60] Kumar P. Gupta G. P. Tripathi R. (2022). PEFL: Deep privacy-encoding-based federated learning framework for smart agriculture. IEEE Micro 42, 33–40. doi: 10.1109/MM.2021.3112476. PMID: 25079929

[B62] Kumar R. Kumar P. Tripathi R. Gupta G. P. Gadekallu T. R. Srivastava G. (2021). SP2F: A secured privacy-preserving framework for smart agricultural unmanned aerial vehicles. Comput. Networks 187, 107819. doi: 10.1016/j.comnet.2021.107819. PMID: 38826717

[B59] Kumar A. Patel V. K. (2023). Classification and identification of disease in potato leaf using hierarchical based deep learning convolutional neural network. Multimedia Tools Appl. 82, 31101–31127. doi: 10.1007/s11042-023-14663-z. PMID: 30311153

[B61] Kumar P. Thanki D. V. Singh S. Nikolovski S. (2020). A new framework for intensification of energy efficiency in commercial and residential use by imposing social, technical and environmental constraints. Sustain. Cities Soc 62, 102400. doi: 10.1016/j.scs.2020.102400. PMID: 38826717

[B64] Lacey H. (2020). The many kinds of objects that technoscientific objects are. Filosofia Unisinos 21, 14–23. doi: 10.4013/fsu.2020.211.02

[B65] Lacueva-Perez F. J. del Hoyo-Alonso R. Labata-Leazaun G. Barriuso-Vargas J. J. Ilarri-Artigas S. (2025). Developing machine learning models from multisourced real-world datasets to enhance smart-farming practices. Comput. Electron. Agric. 231, 110018. doi: 10.1016/j.compag.2025.110018. PMID: 38826717

[B66] Lakhiar I. A. Yan H. Syed T. N. Zhang C. Shaikh S. A. Rakibuzzaman M. . (2025). Soilless agricultural systems: Opportunities, challenges, and applications for enhancing horticultural resilience to climate change and urbanization. Horticulturae 11, 568. doi: 10.3390/horticulturae11060568. PMID: 30654563

[B67] Leal Filho W. Gbaguidi G. J. (2024). Using artificial intelligence in support of climate change adaptation Africa: Potentials and risks. Humanities Soc. Sci. Commun. 11, 1657. doi: 10.1057/s41599-024-04223-7

[B68] Li J. Xu M. Xiang L. Chen D. Zhuang W. Yin X. . (2024). Foundation models in smart agriculture: Basics, opportunities, and challenges. Comput. Electron. Agric. 222, 109032. doi: 10.1016/j.compag.2024.109032. PMID: 38826717

[B69] Licardo J. T. Domjan M. Orehovacki T. (2024). Intelligent robotics-a systematic review of emerging technologies and trends. Electronics 13, 542. doi: 10.3390/electronics13030542. PMID: 30654563

[B70] Lin Y. C. Webster A. J. Scruggs C. E. Bixby R. J. Cadol D. Crossey L. J. . (2025). Fuzzy SETS: Acknowledging multiple membership of elements within social-ecological-technological systems (SETS) theory. Ecol. Soc 30, 22. doi: 10.5751/ES-15764-300122

[B71] Madhuri E. V. Rupali J. S. Sharan S. P. Pooja N. S. Sujatha G. S. Singh D. P. . (2025). Transforming pest management with artificial intelligence technologies: The future of crop protection. J. Crop Health 77, 48. doi: 10.1007/s10343-025-01109-9. PMID: 30311153

[B72] Maraveas C. Asteris P. G. Arvanitis K. G. Bartzanas T. Loukatos D. (2023). Application of bio and nature-inspired algorithms in agricultural engineering. Arch. Comput. Methods Eng. 30, 1979–2012. doi: 10.1007/s11831-022-09857-x. PMID: 30311153

[B73] Maraveas C. Piromalis D. Arvanitis K. G. Bartzanas T. Loukatos D. (2022). Applications of IoT for optimized greenhouse environment and resources management. Comput. Electron. Agric. 198, 106993. doi: 10.1016/j.compag.2022.106993. PMID: 38826717

[B74] McClements D. J. Barrangou R. Hill C. Kokini J. L. Lila M. A. Meyer A. S. . (2021). Building a resilient, sustainable, and healthier food supply through innovation and technology. Annu. Rev. Food. Sci. Technology Annu. Rev. Food. Sci. Technol. 12, 1–28. doi: 10.1146/annurev-food-092220-030824. PMID: 33348992

[B75] Mittal M. Gupta V. Aamash M. Upadhyay T. (2024). Machine learning for pest detection and infestation prediction: A comprehensive review. Wiley Interdiscip. Reviews-Data Min. Knowledge Discov. 14 (5), e1551. doi: 10.1002/widm.1551. PMID: 41531421

[B76] Mmbando G. S. (2025). Harnessing artificial intelligence and remote sensing in climate-smart agriculture: The current strategies needed for enhancing global food security. Cogent Food. Agric. 11, 2454354. doi: 10.1080/23311932.2025.2454354. PMID: 37339054

[B77] Mythen G. (2021). The critical theory of world risk society: A retrospective analysis. Risk Anal. 41, 533–543. doi: 10.1111/risa.13159. PMID: 30170338

[B78] Natarajan K. Chandrasekaran R. (2025). Implementation of artificial intelligence for effective plant disease management. Curr. Sci. 128, 19–20. Available at: https://webofscience.clarivate.cn/wos/woscc/full-record/WOS:001392810200014

[B80] Nayal K. Raut R. Priyadarshinee P. Narkhede B. E. Kazancoglu Y. Narwane V. (2022). Exploring the role of artificial intelligence in managing agricultural supply chain risk to counter the impacts of the COVID-19 pandemic. Int. J. Logistics Manage. 33, 744–772. doi: 10.1108/IJLM-12-2020-0493. PMID: 35579975

[B79] Nayal K. Raut R. D. Queiroz M. M. Yadav V. S. Narkhede B. E. (2023). Are artificial intelligence and machine learning suitable to tackle the COVID-19 impacts? An agriculture supply chain perspective. Int. J. Logistics Manage. 34, 304–335. doi: 10.1108/IJLM-01-2021-0002. PMID: 35579975

[B81] Ntawuruhunga D. Ngowi E. E. Mangi H. O. Salanga R. J. Shikuku K. M. (2023). Climate-smart agroforestry systems and practices: A systematic review of what works, what doesn’t work, and why. For. Policy Econ. 150, 102937. doi: 10.1016/j.forpol.2023.102937. PMID: 38826717

[B82] Oikonomidis A. Catal C. Kassahun A. (2023). Deep learning for crop yield prediction: A systematic literature review. N. Z. J. Crop Hortic. Sci. 51, 1–26. doi: 10.1080/01140671.2022.2032213. PMID: 37339054

[B83] Omotayo A. O. Adediran S. A. Omotoso A. B. Olagunju K. O. Omotayo O. P. (2025). Artificial intelligence in agriculture: Ethics, impact possibilities, and pathways for policy. Comput. Electron. Agric. 239, 110927. doi: 10.1016/j.compag.2025.110927. PMID: 38826717

[B84] Pandey P. C. Pandey M. (2023). Highlighting the role of agriculture and geospatial technology in food security and sustainable development goals. Sustain. Dev. 31, 3175–3195. doi: 10.1002/sd.2600. PMID: 41531421

[B85] Pastor R. Rodriguez P. G. Lecuona A. Cortes J. P. (2025). Smart agri-region and value engineering. Systems 13, 430. doi: 10.3390/systems13060430. PMID: 30654563

[B86] Peters D. P. C. Rivers A. Hatfield J. L. Lemay D. G. Liu S. Basso B. (2020). Harnessing AI to transform agriculture and inform agricultural research. IT Prof. 22, 16–21. doi: 10.1109/MITP.2020.2986124. PMID: 25079929

[B87] Pimenow S. Pimenowa O. Prus P. Niklas A. (2025). The impact of artificial intelligence on the sustainability of regional ecosystems: Current challenges and future prospects. Sustainability 17, 4795. doi: 10.3390/su17114795. PMID: 30654563

[B88] Qi B. Ma X. Yu J. Dong J. Cui X. Zhang X. . (2025). A risk assessment model for the entire rice processing chain based on Kmeans plus plus and extreme learning machine. LWT Food Sci. Technol. 223, 117803. doi: 10.1016/j.lwt.2025.117803. PMID: 38826717

[B89] Quddus M. A. Chowdhury S. Jasper W. Das L. (2025). Enhancing cotton yield prediction with robust deep neural network-based framework. Eur. J. Agron. 170, 127750. doi: 10.1016/j.eja.2025.127750. PMID: 38826717

[B90] Raghuvanshi A. Singh U. K. Sajja G. S. Pallathadka H. Asenso E. Kamal M. . (2022). Intrusion detection using machine learning for risk mitigation in IoT-enabled smart irrigation in smart farming. J. Food Qual. 2022, 3955514. doi: 10.1155/2022/3955514

[B91] Regan A. (2019). “Smart farming” in Ireland: A risk perception study with key governance actors. NJAS Wageningen J. Life Sci. 90–91, 100292. doi: 10.1016/j.njas.2019.02.003. PMID: 38826717

[B92] Reza M. N. Lee K.-H. Karim M. R. Haque M. A. Bicamumakuba E. Dey P. K. . (2025). Trends of soil and solution nutrient sensing for open field and hydroponic cultivation in facilitated smart agriculture. Sensors 25, 453. doi: 10.3390/s25020453. PMID: 39860823 PMC11768686

[B93] Rotz S. Gravely E. Mosby I. Duncan E. Finnis E. Horgan M. . (2019). Automated pastures and the digital divide: How agricultural technologies are shaping labour and rural communities. J. Rural Stud. 68, 112–122. doi: 10.1016/j.jrurstud.2019.01.023. PMID: 38826717

[B94] Rugji J. Erol Z. Tasci F. Musa L. Hamadani A. Gundemir M. G. . (2025). Utilization of AI - reshaping the future of food safety, agriculture and food security—a critical review. Crit. Rev. Food Sci. Nutr. 65, 5136–5180. doi: 10.1080/10408398.2024.2430749. PMID: 39644464

[B95] Sajith G. Srinivas R. Golberg A. Magner J. (2022). Bio-inspired and artificial intelligence enabled hydro-economic model for diversified agricultural management. Agric. Water Manage. 269, 107638. doi: 10.1016/j.agwat.2022.107638. PMID: 38826717

[B96] Salybekova N. Issayev G. Serzhanova A. Mikhailov V. (2024). Utilising artificial intelligence for cultivating decorative plants. Bot. Stud. 65, 39. doi: 10.1186/s40529-024-00445-9. PMID: 39694986 PMC11655720

[B97] Sandbrook C. (2025). Beyond the hype: Navigating the conservation implications of artificial intelligence. Conserv. Lett. 18 (1), e13076. doi: 10.1111/conl.13076. PMID: 40046247

[B98] Schmidt L. Odening M. Schlanstein J. Ritter M. (2022). Exploring the weather-yield nexus with artificial neural networks. Agric. Syst. 196, 103345. doi: 10.1016/j.agsy.2021.103345. PMID: 38826717

[B99] Shehu H. A. Ackley A. Mark M. Eteng O. E. (2025). Artificial intelligence for early detection and management of Tuta absoluta-induced tomato leaf diseases: A systematic review. Eur. J. Agron. 170, 127669. doi: 10.1016/j.eja.2025.127669. PMID: 40464016 PMC12130032

[B100] Shoaib M. Hussain T. Shah B. Ullah I. Shah S. M. Ali F. . (2022). Deep learning-based segmentation and classification of leaf images for detection of tomato plant disease. Front. Plant Sci. 13, 1031748. doi: 10.3389/fpls.2022.1031748. PMID: 36275583 PMC9585275

[B101] Singh S. Goyal M. K. (2023). Enhancing climate resilience in businesses: The role of artificial intelligence. J. Cleaner Prod. 418, 138228. doi: 10.1016/j.jclepro.2023.138228. PMID: 38826717

[B102] Sparrow R. Howard M. Degeling C. (2021). Managing the risks of artificial intelligence in agriculture. Njas-Impact Agric. Life. Sci. 93, 172–196. doi: 10.1080/27685241.2021.2008777. PMID: 37339054

[B103] Stodle K. Flage R. Guikema S. Aven T. (2025). Artificial intelligence for risk analysis-a risk characterization perspective on advances, opportunities, and limitations. Risk Anal. 45, 738–751. doi: 10.1111/risa.14307. PMID: 38600041 PMC12032382

[B104] Su J. Zhu X. Li S. Chen W.-H. (2023). AI meets UAVs: A survey on AI empowered UAV perception systems for precision agriculture. Neurocomputing 518, 242–270. doi: 10.1016/j.neucom.2022.11.020. PMID: 38826717

[B105] Sun Y. Miao Y. Xie Z. Wu R. (2024). Drivers and barriers to digital transformation in agriculture: An evolutionary game analysis based on the experience of China. Agric. Syst. 221, 104136. doi: 10.1016/j.agsy.2024.104136. PMID: 38826717

[B106] Tamberg L. A. Heitzig J. Donges J. F. (2022). A modeler’s guide to studying the resilience of social-technical-environmental systems. Environ. Res. Lett. 17, 055005. doi: 10.1088/1748-9326/ac60d9

[B107] Tawfeek M. A. Yanes N. Jamel L. Aldehim G. Mahmood M. A. (2023). Adaptive deep learning model to enhance smart greenhouse agriculture. Cmc-Computers Materials Continua 77, 2545–2564. doi: 10.32604/cmc.2023.042179

[B108] Tzachor A. Devare M. King B. Avin S. Heigeartaigh S. O. (2022). Responsible artificial intelligence in agriculture requires systemic understanding of risks and externalities. Nat. Mach. Intell. 4, 104–109. doi: 10.1038/s42256-022-00440-4. PMID: 37880705

[B109] Ur Rahim H. Qaswar M. Uddin M. Giannini C. Herrera M. L. Rea G. (2021). Nano-enable materials promoting sustainability and resilience in modern agriculture. Nanomaterials 11, 2068. doi: 10.3390/nano11082068. PMID: 34443899 PMC8398611

[B110] Uyeh D. D. Gebremedhin K. G. Hiablie S. (2023). Perspectives on the strategic importance of digitalization for modernizing African agriculture. Comput. Electron. Agric. 211, 107972. doi: 10.1016/j.compag.2023.107972. PMID: 38826717

[B111] van der Leer J. van Timmeren A. Wandl A. (2018). Social-ecological-technical systems in urban planning for a circular economy: An opportunity for horizontal integration. Archit. Sci. Rev. 61, 298–304. doi: 10.1080/00038628.2018.1505598. PMID: 37339054

[B112] Visser O. Sippel S. R. Thiemann L. (2021). Imprecision farming? Examining the (in)accuracy and risks of digital agriculture. J. Rural Stud. 86, 623–632. doi: 10.1016/j.jrurstud.2021.07.024. PMID: 38826717

[B113] Warner D. Vasseur E. Lefebvre D. M. Lacroix R. (2020). A machine learning based decision aid for lameness in dairy herds using farm-based records. Comput. Electron. Agric. 169, 105193. doi: 10.1016/j.compag.2019.105193. PMID: 38826717

[B114] Xu N. Kang J. Ye Y. Zhang Q. Ke M. Wang Y. . (2022). Machine learning predicts ecological risks of nanoparticles to soil microbial communities. Environ. pollut. 307, 119528. doi: 10.1016/j.envpol.2022.119528. PMID: 35623569

[B116] Yang X. Du J. Jia C. Yang T. Shao S. (2025). Unravelling integrated groundwater management in pollution-prone agricultural cities: A synergistic approach combining probabilistic risk, source apportionment and artificial intelligence. J. Hazard. Mater. 481, 136514. doi: 10.1016/j.jhazmat.2024.136514. PMID: 39566452

[B115] Yang M.-D. Hsu Y.-C. Tseng W.-C. Tseng H.-H. Lai M.-H. (2025). Precision assessment of rice grain moisture content using UAV multispectral imagery and machine learning. Comput. Electron. Agric. 230, 109813. doi: 10.1016/j.compag.2024.109813. PMID: 38826717

[B118] Yang Z. Sono D. (2025). Promoting urban agriculture towards SDGs: An approach of combined assemblage and social-ecological system theories. Habitat Int. 163, 103479. doi: 10.1016/j.habitatint.2025.103479. PMID: 38826717

[B117] Yang X. Zheng X. (2026). Has technical training promoted farmers to adopt green control technologies? Concurrently on the effect of digital platforms. Int. J. Low-Carbon Technol. 21, 1–9. doi: 10.1093/ijlct/ctae260. PMID: 40388063

[B119] Yu H. Tang S. Hamed E. M. Li S. F. Y. Jin Y. Cheng F. (2024). Optimizing the benefit-risk trade-off in nano-agrochemicals through explainable machine learning: Beyond concentration. Environ. Sci. Nano 11, 3374–3389. doi: 10.1039/d4en00213j

[B120] Yuan F. Ospina R. Perumal A. B. Noguchi N. He Y. Liu Y. (2025). Smart agriculture in Asia. Plant Commun. 6, 101377. doi: 10.1016/j.xplc.2025.101377. PMID: 40380769 PMC12281225

[B121] Yuan Y. Sun Y. (2024). Practices, challenges, and future of digital transformation in smallholder agriculture: Insights from a literature review. Agriculture-Basel 14, 2193. doi: 10.3390/agriculture14122193. PMID: 30654563

[B122] Yuan Y. Sun Y. (2025). The values, challenges, and strategies of AI in empowering sustainable livelihoods for farmers. Front. Sustain. Food Syst. 9, 1716572. doi: 10.3389/fsufs.2025.1716572

[B123] Zewdu D. Krishnan C. M. Raj P. P. N. Arlikatti S. McAleavy T. (2025). Climate-smart innovation practices and sustainable rural livelihoods: A systematic literature review. Technol. Soc. 82, 102914. doi: 10.1016/j.techsoc.2025.102914. PMID: 38826717

[B124] Zogaan W. A. Ajabnoor N. Salamai A. A. (2025). Leveraging deep learning for risk prediction and resilience in supply chains: Insights from critical industries. J. Big Data 12, 94. doi: 10.1186/s40537-025-01143-4. PMID: 38164791

[B125] Zscheischler J. Brunsch R. Rogga S. Scholz R. W. (2022). Perceived risks and vulnerabilities of employing digitalization and digital data in agriculture-socially robust orientations from a transdisciplinary process. J. Cleaner Prod. 358, 132034. doi: 10.1016/j.jclepro.2022.132034. PMID: 38826717

